# A Review on Micromixers, Microdroplet Generators and Their Integration

**DOI:** 10.3390/mi17080963

**Published:** 2026-08-15

**Authors:** Wang He, Ling Zhang, Lei Wu, Yushan Chen, Tingting Chen

**Affiliations:** 1 College of Mechanical and Electrical Engineering, Xichang University, Xichang 615000, China; 2 Tribology Research Institute, School of Mechanical Engineering, Southwest Jiaotong University, Chengdu 610031, China; 3 The 10th Research Institute of China Electronics Technology Corporation, Chengdu 610036, China; 4 State Key Laboratory of Tribology, Tsinghua University, Beijing 100084, China

**Keywords:** micromixers, microdroplet generators, microfluidic device, microfluidic integration, droplet microreactors

## Abstract

High-efficiency mixing and precise droplet generation are essential for the broad application of microfluidic chips in biology, chemistry, and medicine, with integrated micromixer and microdroplet generator systems playing an increasingly important role in diagnostics and drug detection. This review offers a comprehensive and systematic overview of micromixers and microdroplet generators, covering their classification, working principles, performance characterization, and integration strategies. Passive and active micromixers, the latter employing pressure, electric, acoustic, magnetic, and thermal fields to enhance mixing, are summarized with a critical discussion of their advantages, limitations, and structural optimization. Various droplet generation methods, including crossflow, flow focusing, coflow, step emulsification, and active techniques, are examined alongside key parameters affecting droplet size, monodispersity, and generation frequency. Performance characterization metrics for both components, including mixing efficiency, mixing time, pressure drop, droplet size distribution, generation frequency, and stability, are also discussed. The review then focuses on integration, emphasizing two primary architectural strategies: mixing reagents before encapsulation and inducing mixing within droplets after formation. Synergistic applications in single cell analysis, materials synthesis, and drug screening are presented. Notably, unlike previous reviews that treat micromixers or droplet generators separately, this review uniquely emphasizes architectural integration and critically evaluates the associated trade-offs. Current challenges and future directions, including material selection, fabrication techniques, and practical application-oriented considerations, are also addressed.

## 1. Introduction

Microfluidics, the technology of manipulating small volumes of fluids within microfabricated channels, has revolutionized diverse fields, including chemistry, biology, medicine, and materials science [1,2,3,4,5,6]. The miniaturization of microfluidic devices provides many advantages over conventional systems, such as reduced sample and reagent consumption, improved analytical sensitivity, and increased throughput [3,7,8,9,10,11]. Micro-mixers and micro-droplet generators, as integral components of microfluidic chip systems, enable the precise control of fluids in microchannels and offer unique features for a wide range of applications [12,13,14,15]. To ensure reproducibility, we systematically searched Web of Science, Scopus, and PubMed using keywords such as micromixer, droplet generation, and microfluidic integration. The search mainly covered January 2015 to April 2026. We included peer-reviewed English articles reporting quantitative performance data. From 1247 initial records, 368 articles were selected after title/abstract screening and full-text assessment.

Efficient mixing is essential for many applications, including chemical reactions, bioassays, sample preparation, and materials synthesis [16,17,18,19]. However, the laminar flow regime that typically prevails in microfluidic channels, characterized by low Reynolds numbers, poses a challenge for effective mixing. In laminar flow, fluid mixing relies on slow molecular diffusion, which can be prohibitively time-consuming for many applications [20]. Therefore, specialized channel geometries or external energy inputs are required to enhance mixing performance. Micromixers can be broadly categorized into two main types: passive and active [21]. Passive micromixers leverage intricate channel geometries to enhance mixing without external power, but their efficiency is often moderate and highly dependent on flow conditions. In contrast, active micromixers utilize external energy sources—such as acoustic, electric, or thermal fields—to induce powerful fluid motion, enabling rapid and controllable mixing [22,23,24,25]. This superior performance, however, comes at the cost of increased device complexity, power requirements, and fabrication expense. Consequently, the selection of an optimal micromixer is a critical design decision based on a trade-off between performance requirements and operational constraints.

Microdroplet generators are another critical component in microfluidic systems, enabling the generation of strongly monodisperse droplets with precisely controlled sizes, compositions, and generation frequencies [26,27]. These droplets are employed as miniaturized reaction vessels to provide isolated environments for chemical and biological assays, synthesizing materials, and encapsulating cells or biomolecules. Droplet-based microfluidics has emerged as a powerful tool for high-throughput screening, single-cell analysis, drug discovery, and materials science [28]. Similarly to micromixers, microdroplet generators can be classified into passive and active types [29]. Passive droplet generators rely on the interplay of interfacial tension, viscous forces, and channel geometry to generate droplets, while active methods utilize external forces, such as electric fields or acoustic waves, to control droplet formation [30,31,32]. Precise control over droplet size, monodispersity, and generation frequency is crucial for many applications, as these parameters can significantly influence experimental results.

**Figure 1:**
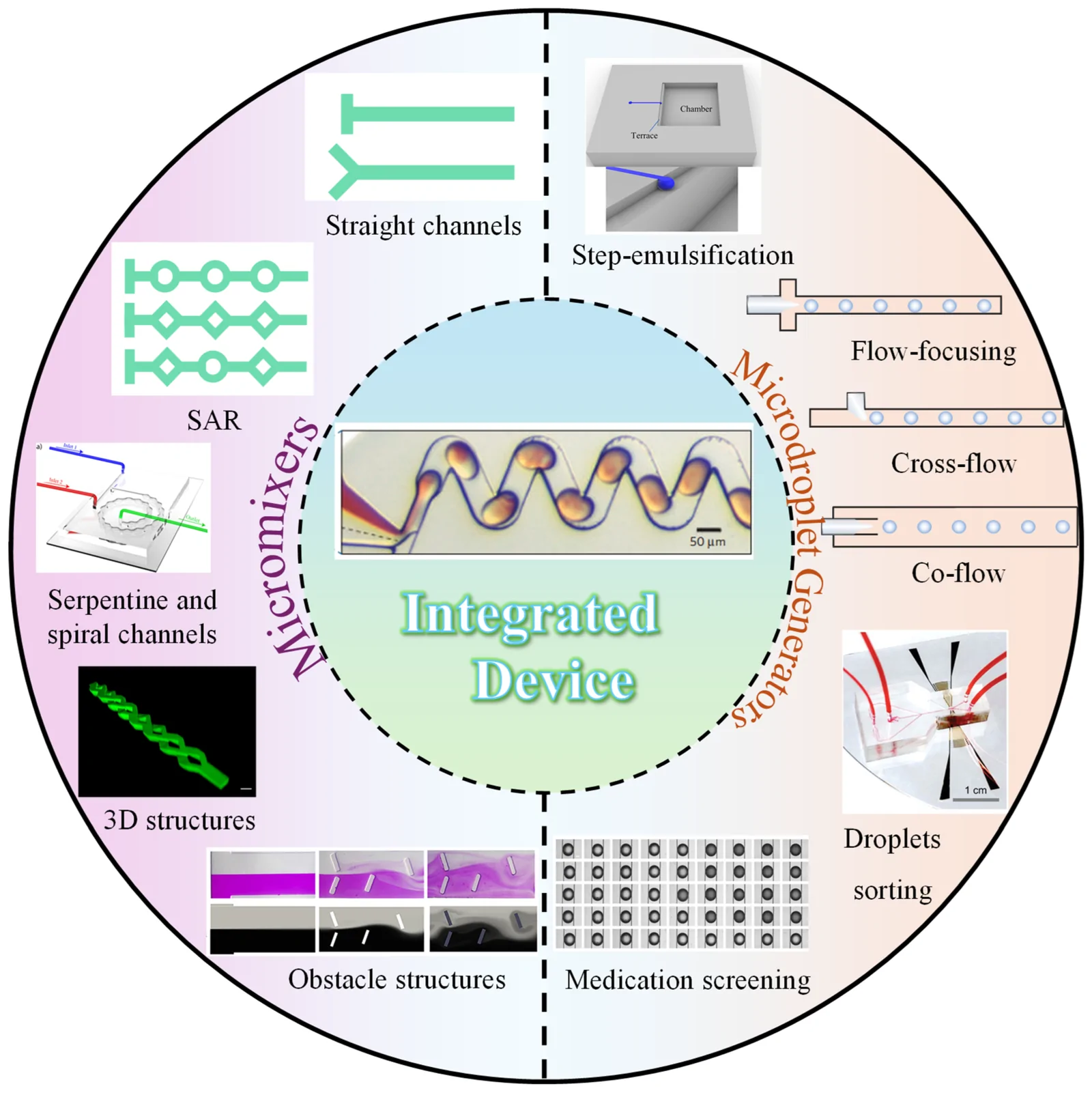
An overview of micromixers, micro-droplet generators and their integrated device applications. The figure of serpentine and spiral channels is reproduced with permission from Bahrami, D.; Bayareh, M, Chemical Engineering and Technology (CET); published by Wiley 2022 [36].

The integration of micromixers and microdroplet generators within microfluidic chips enables synergistic applications (Figure 1). For example, incorporating a micromixer upstream of a droplet generator can facilitate rapid mixing of reagents within droplets, enhancing reaction kinetics and improving assay performance. Furthermore, the combination of micromixers and droplet generators can be utilized for high-throughput screening of chemical and biological reactions, generating large libraries of droplets with varying compositions and analyzing their contents in parallel [33]. Droplet-based microfluidics has also become an indispensable tool for single-cell analysis, allowing the isolation and manipulation of individual cells within droplets, followed by downstream analysis of their genomic, transcriptomic, and proteomic profiles [34].

Despite the remarkable advancements in micromixer and microdroplet generator technologies, several challenges remain to be addressed. The development of novel designs to improve mixing efficiency at low Reynolds numbers remains an active area of research. Furthermore, reducing the cost and complexity of fabrication processes is essential for wider adoption of microfluidic technologies. Future research directions include the development of integrated microfluidic platforms combining multiple functionalities, such as droplet generation, mixing, sorting, and detection, for automated and high-throughput analysis. The exploration of new materials and fabrication techniques, such as 3D printing and micromachining, will further expand the capabilities and applications of micromixers and microdroplet generators [35]. The ongoing development of these microfluidic components promises to drive further innovation and accelerate discoveries across a broad range of scientific and technological disciplines.

## 2. Micromixers

### 2.1. Classification and Working Principles

Micromixers, as essential components in microfluidic chips, are designed to achieve the rapid mixing of multiple fluids. They play significant roles in applications such as chemical reactions, bioanalysis, and materials synthesis [15,37]. In microchannels, the low Reynolds number typically results in a laminar flow regime where mixing relies on slow molecular diffusion, leading to inefficiency [38]. Therefore, specialized channel geometries and external energy inputs are required to improve mixing performance. Depending on the use of external energy sources, micromixers are categorized into passive and active types [21]. 

#### 2.1.1. Passive Micromixers

Passive micromixers utilize the fluid kinetic energy and channel architecture designed to enhance mixing, offering advantages such as structural simplicity, easy fabrication, and integration. However, their limitations include relatively low mixing efficiency and dependence on flow velocity. The fundamental mechanism relies on molecular diffusion under laminar flow conditions. This method is only effective at low flow rates with extended channel lengths, leading to prolonged mixing times, as observed in spiral and serpentine channels. Curved microchannels generate counter-rotating Dean vortices due to centrifugal forces, enhancing mixing [39]. Spiral and meandering channels leverage this effect, with the Dean number determining vortex strength and mixing performance. Serpentine designs increase diffusion by elongating the flow path and contact area but may induce higher pressure drops [40]. In pressure-driven laminar flows, the parabolic velocity profile induces solute dispersion along the flow direction. Taylor dispersion increases the effective diffusion coefficient, which is particularly effective at low Reynolds numbers. Some geometries (e.g., obstacles, grooves, and ridges) disrupt the laminar flow to generate transverse flows and vortices, significantly improving mixing efficiency [41,42].

#### 2.1.2. Active Micromixers

Active micromixers require external energy to drive fluid and enhance mixing performance. These devices demonstrate superior mixing efficiency with reduced mixing time compared to passive counterparts, albeit with increased structural complexity, fabrication challenges, and higher costs. Active micromixers generally rely on externally applied energy fields, such as electric, acoustic, magnetic, or thermal fields, to actively perturb the flow and enhance mixing [43,44,45,46]. In contrast, pressure-driven flow, which is the standard driving mode for most microfluidic systems, serves as the basis for both passive and active devices but does not itself constitute an active mixing mechanism. The comparative analysis reveals that while active micromixers achieve excellent mixing performance, their application is constrained by fluid property requirements. This limitation highlights the complementary nature with passive micromixers employing asymmetric structures for wider applicability.

**Figure 2:**
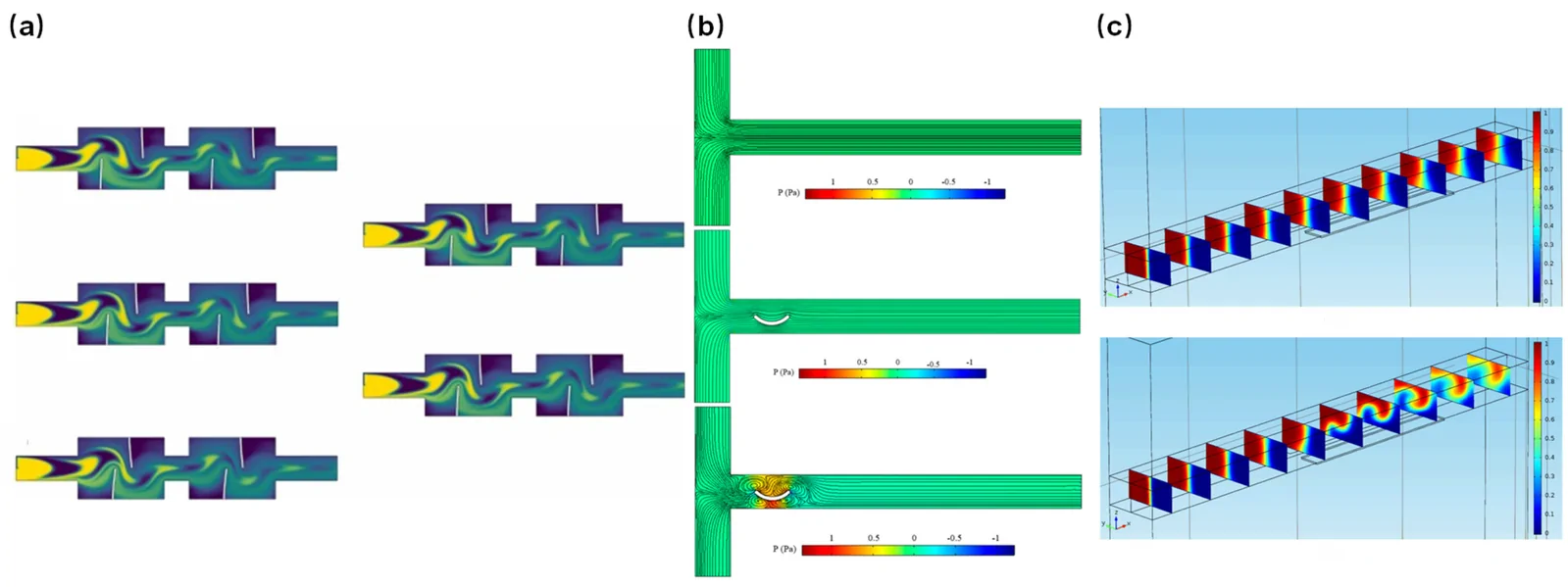
Active micromixers with different structures and their numerical simulation. (**a**) Dye mass fraction distribution under different inlet conditions. Reprinted with permission from Jahangirifard et al., Chem. Eng. Res. Des. 208, 588–598 (2024) [45]. Copyright 2024 Elsevier. (**b**) Flow pattern and concentration distribution inside the micromixer for cases of without mounting the flap, with mounting a flap, and with mounting a fully conductive flap. Reprinted from Goodarzi et al., Symmetry 13, 915 (2021) [25], an open access article distributed under the terms of the Creative Commons CC BY license. Copyright 2021 by the authors. (**c**) Observations of fluid mixing with different microheater locations, strengths and structures. Reprinted with permission from Zhang et al., Adv. Mech. Eng. 8, 1–10 (2016) [23]. Copyright 2016 SAGE Publications.

Contrary to passive devices that rely on channel geometry optimization, active implementations employ controlled energy inputs to manipulate fluid dynamics at microscale dimensions. As shown in Figure 2a, Shayan Jahangirifard et al. developed an innovative micromixer based on deformable baffles coupled with pulsatile flow in microchannels, where mixing enhancement is achieved by the combination of flexible obstacles and oscillatory flow conditions [45]. An active piezoelectric micromixer based on multiple sharp-edged acoustic flow modes was reported by Zhang et al. and the mixing efficiency was verified by the iodide–iodate reaction visualization. The acoustic vortices generated by its sharp-edged structures synergize with the main flow to form three-dimensional chaotic convection, which drastically reduces the mixing time from the baseline value of 0.28 s to 0.03 s [44]. A novel active micromixer based on the jet countercurrent mechanism is proposed in a study to realize efficient mixing by constructing a jet countercurrent zone and an annular liquid film zone [47]. As shown in Figure 2c, Feng Zhang et al. numerically investigated a novel AC electrothermal micromixer with thin film resistive heaters, where mixing enhancement is achieved by the external heating-induced temperature gradient that generates flow body forces and chaotic stretching and folding of fluid material lines, with the effects of heater size and location on mixing efficiency also examined [23].

Active micromixers offset the shortcomings of passive mixers in terms of mixing length, time, pressure drop, etc. Recent advances in microfabrication and field control algorithms continue to address current limitations, particularly in biocompatibility and energy efficiency. The optimal mixer selection ultimately depends on specific application requirements regarding throughput, fluid properties, and operational constraints.

### 2.2. Structural Optimization

The mixing performance of micromixers is closely related to their structural design. Optimizing geometric configurations, dimensions, and fluid control can enhance mixing efficiency while reducing mixing time and pressure drop. This section discusses the impact of various structural design parameters on micromixer performance in detail. This subsection focuses primarily on structural optimization strategies for passive micromixers, including geometric configurations, channel dimensions, and fluid parameters. Active micromixers, by contrast, are optimized mainly through tuning external field parameters such as frequency, amplitude, and voltage, which have already been discussed in Section 2.1.2 and are therefore not reiterated here. Accordingly, the following analysis concentrates on passive designs, while the performance trade-offs discussed are equally relevant for guiding the selection and improvement in both passive and active devices.

Before discussing structural optimization, we first define the three key performance metrics used throughout this section. Mixing efficiency, quantified by the mixing index, measures the uniformity of fluid distribution across the channel cross-section, with unity indicating complete homogenization. Mixing time is the duration required to reach a specified mixing level, typically 90% or 95%. Pressure drop reflects the hydrodynamic resistance and determines the required driving force. These metrics are interdependent, as enhancements in mixing efficiency often come at the cost of increased pressure drop.

#### 2.2.1. Geometry

Straight channels: Straight channels represent the simplest micromixer structures. However, due to low Reynolds number laminar flow constraints, mixing primarily relies on diffusion, resulting in extremely low efficiency [48,49]. This structure is rarely used on its own because of its dependence on molecular diffusion and low mixing efficiency. Optimization has been directed mainly towards the introduction of other structures, such as barriers or ridges, to enhance convective mixing [50]. For straight channels, the shape of the intersection of the two fluids in the channel also influences the mixing efficiency. In a study, chaotic flow-driven mixing in T- and V-shaped micromixers with high Reynolds numbers was investigated using fluorescence microscopy and numerical analysis. The results show that the asymmetry in the V-mixer enhances the mixing homogeneity, while the T-shaped mixer requires a higher Reynolds number for complete mixing [48].

**Figure 3:**
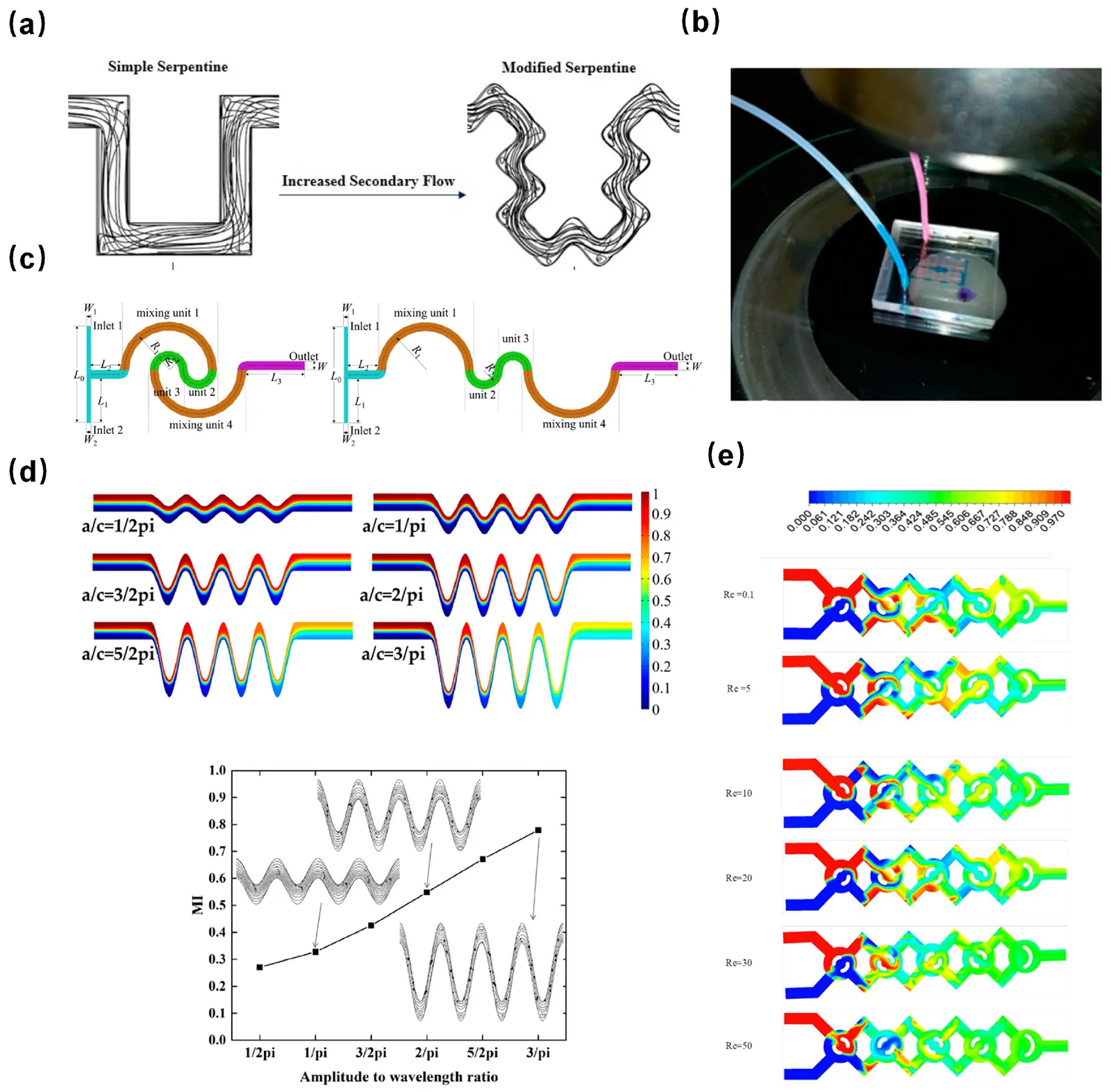
Different spiral and serpentine channels (**a**) Spiral micromixer with sinusoidal channel walls and the geometrical dimension. Reprinted from Javaid et al., Micromachines 9, 8 (2018) [40], an open access article distributed under the terms of the Creative Commons CC BY license. Copyright 2018 by the authors. (**b**) The PMMA micromixers of spiral and serpentine structures. Reprinted from Zoupanou et al., Micromachines 12, 104 (2021) [51], an open access article distributed under the terms of the Creative Commons CC BY license. Copyright 2021 by the authors. (**c**) Schematic diagram of the spiral and serpentine micromixers. Reprinted from Qin et al., Micromachines 16, 1016 (2025) [52], an open access article distributed under the terms of the Creative Commons CC BY license. Copyright 2025 by the authors. (**d**) Distribution of concentration inside the horizontal mid-plane and the vertical plane in the six sinusoidal microchannels. Reprinted from Chen et al., Micromachines 13, 1933 (2022) [53], an open access article distributed under the terms of the Creative Commons CC BY license. Copyright 2022 by the authors. (**e**) Mass fraction distribution of fluid in the raccoon and serpentine micromixer. Reprinted from Mahammedi et al., Energies 17, 3248 (2024) [54], an open access article distributed under the terms of the Creative Commons CC BY license. Copyright 2024 by the authors.

As shown in Figure 3a, Muhammad Usman Javaid et al., designed a serpentine-shaped passive micromixer with sinusoidal side walls that can increase the mixing index in the low to medium Reynolds number range. The proposed sinusoidal walled serpentine channel achieved a comparatively higher mixing index than a simple serpentine channel with a relatively lower or similar mixing cost, and higher mixing efficiency was obtained by increasing the amplitude-to-wavelength (A/λ) ratio [40]. In the work of Zoupanou et al., the mixing efficiencies of spiral and serpentine micromixers were compared by numerical simulations and experimental studies over a wide range of Reynolds numbers (Figure 3b) [51]. In this study, it was found that the structure of Dean vortices plays an important role in material transfer during the fluid mixing process.

Many researchers have used numerical simulations to compare channels with different parameter structures. As shown in Figure 3, the effects of different amplitudes, periods and phase differences in the sinusoidal channel on the mixing efficiency are compared [52,55]. There are many other types of serpentine micromixers where the mixing process is chaotic advection due to Dean vortices, stretching flow and asymmetric vortices. Spiral channels leverage Dean vortex effects to enhance transverse mixing [56]. The curvature radius and number of spiral turns influence Dean vortex intensity and mixing efficiency [57]. Performance can be optimized by adjusting curvature, introducing obstacles, or modifying cross-sectional shapes. Zigzag channels, similar to serpentine designs, induce stronger chaotic convection at higher Reynolds numbers through abrupt directional changes (e.g., right-angle turns), improving mixing efficiency [40,58]. When designing serpentine and spiral micromixers of different structures, researchers should aim not only to improve their mixing efficiency but should also consider the pressure drop as a key factor. In evaluating the pressure drop of spiral and serpentine micromixers, a study has demonstrated through numerical simulation that the pressure drop of spiral micromixers is greater than that of serpentine micromixers, using mixing efficiency and pressure drop as important parameters. At higher Reynolds numbers, the serpentine micromixer provides nearly the same mixing efficiency and outperforms the spiral micromixer at relatively low pressure drops [59].

**Figure 4:**
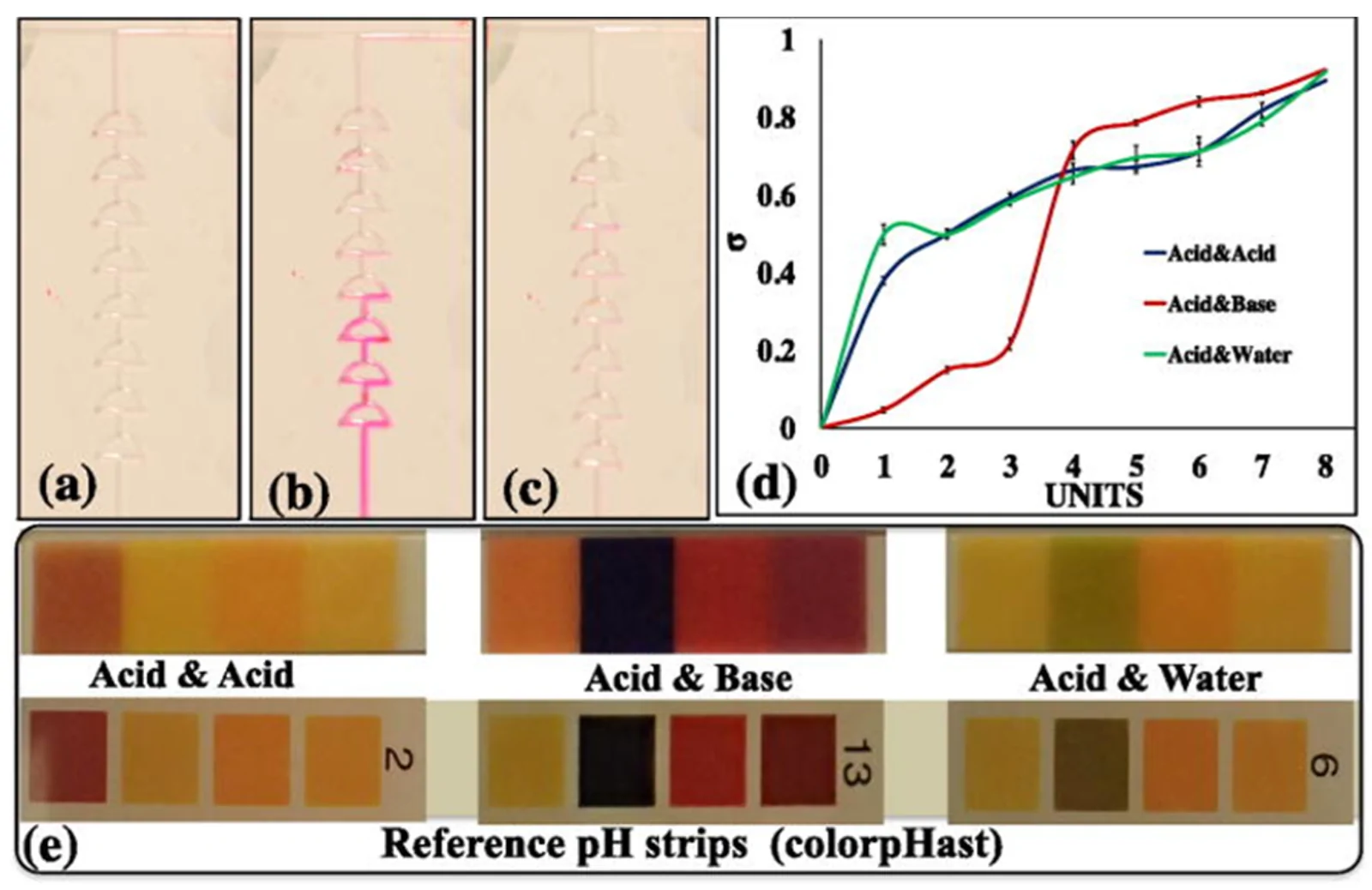
Concentration/velocity profiles on three SAR micromixers. (**a**) Mixing of acid and acid; (**b**) mixing of acid and base; (**c**) mixing of acid and water; (**d**) plots of gray values of mixing in each case; and (**e**) change in pH compared with commercially available pH strips. Reprinted from Sivashankar et al., Biomicrofluidics 10, 034120 (2016) [61], an open access article distributed under the terms of the Creative Commons CC BY license. Copyright 2016 by the authors.

Split-and-recombine (SAR): SAR structures repeatedly divide and recombine fluid, creating interlaced thin layers that reduce diffusion distances and enhance mixing efficiency [60]. As shown in Figure 4, Shilpa Sivashankar et al. designed a new “twisted” 3D passive microfluidic mixer fabricated by laser writing and microfabrication techniques, which combines splitting/recombining and chaotic advection mechanisms, and mixing enhancement is achieved by the three-dimensional serpentine channel path and the lamination of mixing units arranged in a two-layered serial configuration [61]. This structure is more complex compared to other structures of micromixers. The channels used for mixing are generally longer in order to repeatedly split and recombine the fluid, which in turn leads to higher pressure drop.

**Figure 5:**
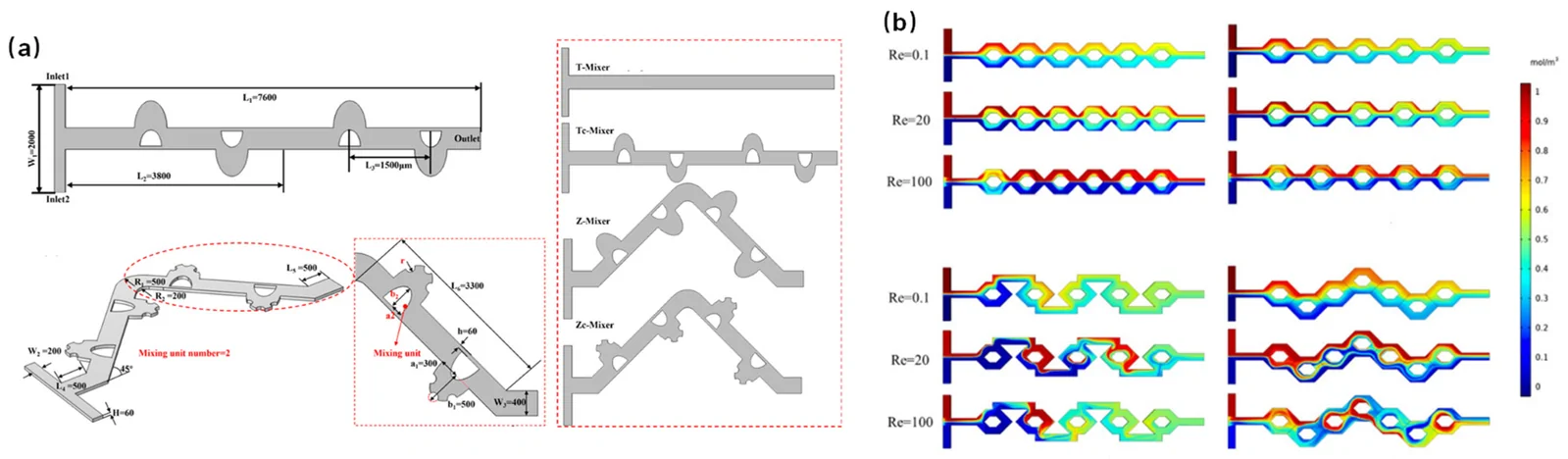
Some obstacle structures in the channel. (**a**) Some micromixers with obstacles contain diagonal obstacles, grooved microstructures and diagonal obstacles. Reprinted from Yuan et al., Micromachines 17, 190 (2026) [66], an open access article distributed under the terms of the Creative Commons CC BY license. Copyright 2026 by the authors. (**b**) The designed micromixer schematic and the experimental concentration distribution. Reprinted from Nishu and Samad, Heliyon 9, e14745 (2023) [64], an open access article distributed under the terms of the Creative Commons CC BY-NC-ND license. Copyright 2023 by the authors.

Obstacle structures: Introducing obstacles including cylindrical obstructions, triangular obstructions, fan-shaped, and hexagonal obstructions, or modifying inlet channel angles can improve mixing [62,63]. Obstacle-embedded channels disrupt streamlines and promote transverse mixing via chaotic advection. These baffles cause secondary flows to grow in the channel, thus enhancing its mixing index (Figure 5) [64,65]. The performance of such devices is critically dependent on the geometric parameters of the obstacles, including their shape, size, spacing, and arrangement [64,65,66]. It is worth noting that although this method can enhance the mixing efficiency to a certain extent, this improvement often comes at the cost of an increased pressure drop.

**Figure 6:**
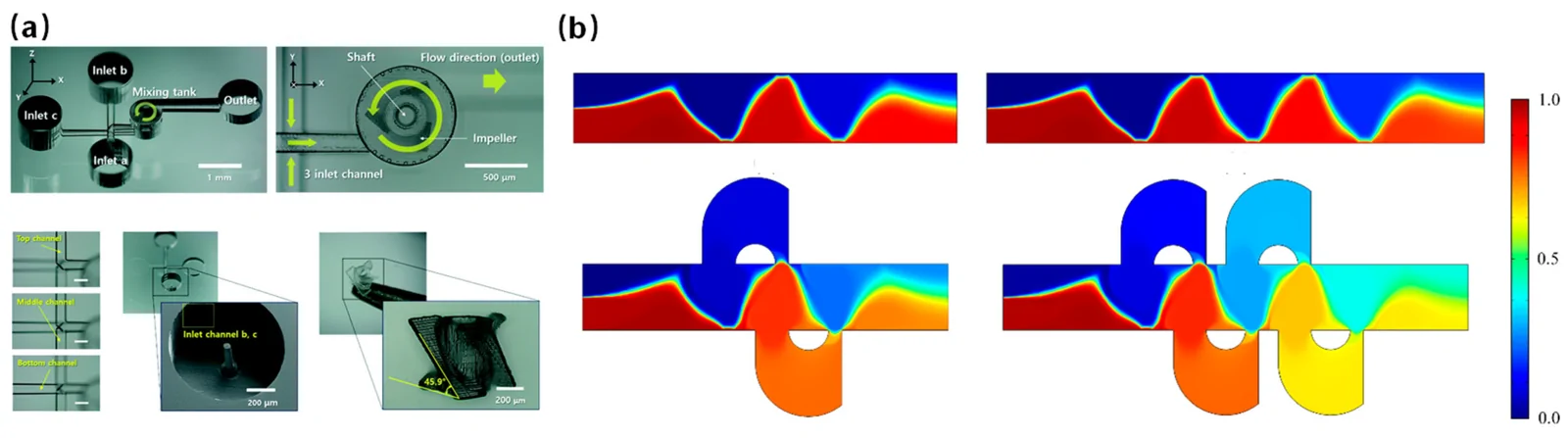
Chamber-integrated channels. (**a**) Images of multilevel entryways, single mixing tanks and single exits. Adapted and used with permission from Kim et al., Lab Chip 20, 4474–4485 (2020) [67]. Copyright 2020 Royal Society of Chemistry, permission conveyed through Copyright Clearance Center, Inc. (**b**) Fluid distribution in a geometrically scalable active micromixer. Reprinted from Chen et al., Micromachines 8, 105 (2017) [68], an open access article distributed under the terms of the Creative Commons CC BY license. Copyright 2017 by the authors.

Chamber-integrated channels: Mixing in chamber-integrated channels is achieved by generating recirculation zones and vortices, and their efficiency can often be enhanced by optimizing chamber geometry and position. Chamber-integrated channels often incorporate additional physical fields to enhance mixing efficiency. As shown in Figure 6, the chamber system contains active elements that can utilize various external dynamics to cause fluid perturbation or incorporate fluid channels with obstructions to increase diffusion [67,68]. To have enough space to mix the liquid, this kind of micromixer usually comes with a larger volume of chamber, thus resulting in a higher mixing efficiency and a lower pressure drop.

**Figure 7:**
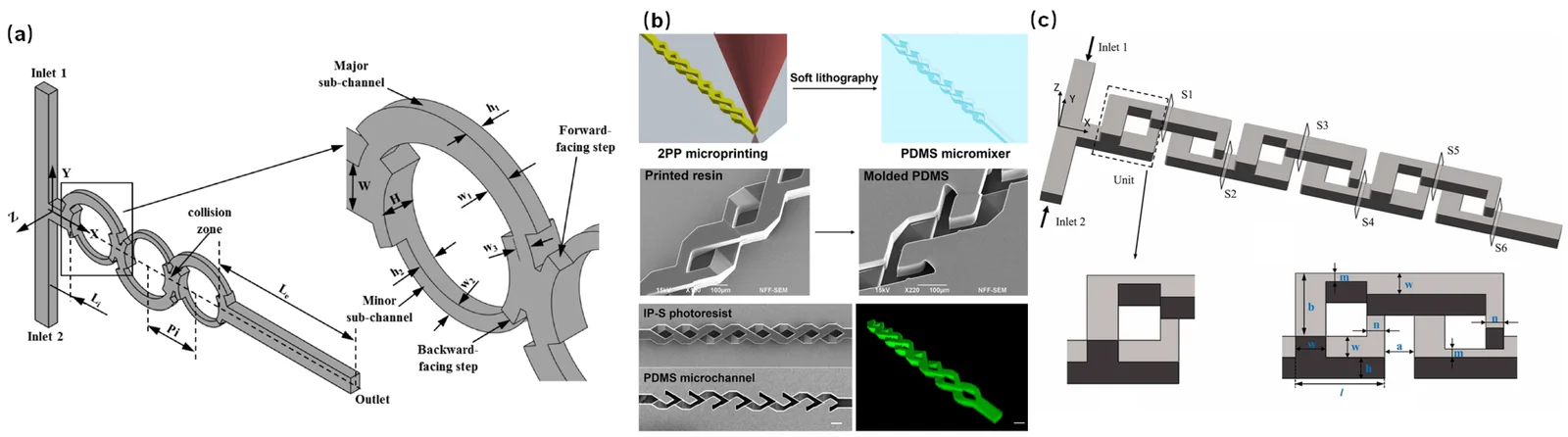
Various micromixers with 3D structures. (**a**) Schematic diagram of a micromixer with a circular mixing unit. Reprinted from Raza et al., Micromachines 10, 711 (2019) [69], an open access article distributed under the terms of the Creative Commons CC BY license. Copyright 2019 by the authors. (**b**) Fabrication of 3D PDMS micromixer and its SEM image. Reprinted with permission from Wang et al., Anal. Chem. 95, 8850–8858 (2023) [70]. Copyright 2023 American Chemical Society. (**c**) Three-dimensional view of the integrated vortex rectangular micromixer. Reprinted from Jiao et al., J. Micromech. Microeng. 32, 075007 (2022) [71]. Copyright 2022 IOP Publishing.

Three-dimensional structures: Compared to the micromixer with 2D structures, the micromixer with Three-dimensional structures has a greater advantage in fluid mixing, but machining of fine 3D structures remains a challenge. 3D structures can enhance mixing and minimize diffusion distances through multilayer structures and varying paths [69]. Many of the 3D micromixer structures are also designed similarly to the split-and-recombine structure but with an additional dimension [70,71,72]. As shown in Figure 7, a polydimethylsiloxane (PDMS) micromixer optimized according to the SAR structure is reported to enable efficient and fast mixing of fluids at low Reynolds numbers [70]. In addition, 3D spiral structures (e.g., double helices), as typical 3D micromixers, can amplify Dean vortices and enable interlayer mixing. Bridge structures connect 3D components to facilitate cross-layer fluid exchange [73].

#### 2.2.2. Structural Dimensions

Microfluidic channel dimensions significantly influence fluid mixing. Reducing channel width and height decreases diffusion distances and increases surface area-to-volume ratios, thereby enhancing mixing. However, smaller channels are susceptible to clogging and exhibit increased pressure drop. Introducing obstacles within the channel further promotes mixing by disrupting the flow field. Larger obstacles generate stronger vortices and consequently improve convective mixing, but at the cost of higher pressure drop. While smaller channels favor laminar diffusive mixing at low Reynolds numbers, they inherently increase flow resistance and pressure drop. The size and spacing of obstacles dictate the extent of flow disturbance and vortex generation. A larger obstacle blockage ratio intensifies the resultant vortices, but also elevates pressure drop. Similarly, smaller radius of curvature in curved channels enhances Dean vortices, improving mixing efficiency while simultaneously increasing pressure drop. In contraction-expansion channels, the amplitude and wavelength of the geometric variations determine the strength and effectiveness of the expansion vortices generated. Optimizing these parameters requires a careful balance between mixing performance and pressure drop considerations. The relationship between a smaller radius of curvature leading to stronger Dean vortices and higher mixing efficiency, coupled with increased pressure drop, highlights this trade-off [74].

#### 2.2.3. Fluid Parameter

Micromixer performance is highly dependent on fluid characteristics. Residence time and convective mixing are governed by flow velocity, with diffusion dominating at low Reynolds numbers (Re) and convection at high Re. Optimal Re ranges vary between micromixer designs, and inlet flow rate ratios significantly affect interfacial area and mixing homogeneity, particularly in multi-inlet devices where these ratios are critical. Furthermore, stratified inlet flows can enhance mixing by expanding the fluid interface.

Microfluidic flows are predominantly laminar due to the predominance of viscous forces. At very low Re (<<1), inertial forces become negligible, approaching Stokes flow for steady, incompressible Newtonian fluids. In the intermediate Re regime, weak inertial effects permit the utilization of secondary flows, such as Dean vortices, to enhance microscale fluid control capabilities. Nonlinearities arising from non-Newtonian fluid properties, multiphase flows, elastic channel walls, and stimuli-responsive materials further enrich the flow dynamics. These nonlinearities can be exploited to design a variety of microfluidic components, including mixers, valves, oscillators, flow stabilizers, and auto-regulators, thereby expanding the capabilities of nonlinear microfluidics [75].

While the laminar, stable, and reversible flows characteristic of conventional linear microfluidics (dominated by viscous forces and negligible inertia) are beneficial for applications such as diffusive separation and precision material synthesis [74,75,76,77,78,79], the lack of significant inertial forces limits certain functionalities. For example, transverse transport in laminar microflows relies heavily on diffusion and conduction, which can be inefficient despite the increased surface area-to-volume ratio. This inherent stability also hinders integrated flow control, often requiring external control systems [80,81,82]. Consequently, strategies for manipulating and destabilizing these linear flows are highly sought after.

In a research work, numerical methods were used to study the effect of transverse structures on laminar flow, in particular the generation of Dean vortices. The results showed that the formation of eddies depends on the velocity difference between the lateral structure and the main channel. The velocity component perpendicular to the main channel and the minimum flow velocity variation along the channel optimized the generation of these secondary flows [83].

#### 2.2.4. Selection of Micromixers

The optimal micromixer design is application-dependent, governed by operational constraints and performance metrics. Optimizing one performance metric, such as mixing speed, often compromises another, like pressure drop or biocompatibility. Therefore, careful consideration of these factors is crucial during the structural design.

When working with live cells, proteins, or other sensitive biomolecules, the primary design considerations are minimizing shear stress and ensuring biocompatibility. High shear forces can induce cell damage or lysis and protein denaturation, compromising experimental results. Consequently, designs featuring sharp obstacles, abrupt contractions, or those requiring high flow velocities should be avoided. Instead, passive designs like serpentine channels, which induce mixing through gentle Dean vortices, are often preferred. Alternatively, low-power active mixers, such as those based on acoustic streaming, can provide rapid and efficient mixing while imparting minimal mechanical stress on the biological samples, making them an excellent choice for delicate applications [84]. The choice of material, typically PDMS or other biocompatible polymers, is also paramount.

In applications such as rapid chemical synthesis or the controlled formulation of nanoparticles, the priority is typically the mixing speed. Rapid and complete homogenization of reagents is essential to ensure uniform reaction kinetics and to produce monodisperse products. In this context, parameters like pressure drop and shear stress are often secondary concerns. Therefore, more aggressive passive designs, including those with obstacle-based structures or complex 3D geometries, are highly effective as they generate strong transverse flows and rapid vortexing [85].

The design of micromixers for point-of-care testing (POCT) applications is governed by many constraints, such as low power consumption, operational simplicity, and low manufacturing cost. This type of device typically relies on simple portable drive mechanisms, such as syringe pumps or even capillary action. This necessitates designs with a low pressure drop to minimize the required driving force. Furthermore, ease of mass production and integration favors simpler geometries [86]. For these reasons, simple passive micromixers, such as T-mixers or short serpentine channels, are a suitable choice, as they offer adequate mixing for many diagnostic assays without the complexity and power requirements of active systems or high-resistance passive designs [87].

### 2.3. Performance Characterization

The performance of a micromixer is usually characterized by metrics such as mixing efficiency, mixing time, and pressure drop. These metrics are quantified through various approaches, reflecting the performance of the micromixer from different perspectives. The selection of appropriate performance metrics is essential to evaluate and compare different micromixer designs.

#### 2.3.1. Mixing Efficiency

Mixing efficiency, as an important measure of micromixer performance, has been extensively reported in the literature [88,89]. Ideally, the concentration of the two fluids should be uniformly distributed after complete mixing. Mixing efficiency is usually expressed as the mixing index (MI), for which the closer the MI value is to unity, the higher the mixing homogeneity, while a value of 0 indicates no mixing. This approach evaluates mixing uniformity based on the variance of the concentration across the channel cross-section.

(1) $$\begin{array}{c} MI=1-\sqrt{\frac{I^{2}}{I_{max}}} \end{array}$$

Here, *I*^2^ is the variance of the concentration in the channel cross section and Imax is the maximum variance of the concentration in the completely unmixed state. In this review, we consistently adopt the variance-based mixing index defined by Equation (1) as our reference standard for discussing reported mixing performances. However, we acknowledge that the original cited literature does not always employ this exact definition. Some studies quantify mixing using grayscale intensity variance, while others apply standard deviation-based normalization or alternative formulations. This variability across research groups should be borne in mind when comparing numerical values reported by different studies. Unless otherwise stated, all mixing index values cited in this review are based on the definitions provided in the original references.

#### 2.3.2. Mixing Time

Mixing time is the time it takes to reach a specific mixing efficiency, usually 90% or 95% mixing efficiency. A shorter mixing time indicates faster mixing. Methods for measuring mixing time include those based on image analysis and chemical reactions. Mixing times can be determined by recording an image sequence of the mixing process and analyzing the MI at different time points. Using chemical reactions that are sensitive to mixing, such as the iodide–iodate reaction, enables the measurement of mixing time.

#### 2.3.3. Pressure Drop

Pressure drop is the pressure loss that occurs when fluid flows through a micromixer and reflects the resistance the micromixer exhibits against the fluid. A smaller pressure drop results in lower flow resistance and a lower required driving pressure, which facilitates energy savings and higher throughput. The pressure difference between the inlet and outlet of the micromixer can be measured using a pressure transducer. Fluid flow can also be simulated using computational fluid dynamics (CFD) software and the pressure drop calculated. Consideration of pressure drop is essential when designing a micromixer structure; otherwise, excessive pressure drop can lead to device leakage [90]. Therefore, a trade-off between mixing performance and pressure drop must be carefully balanced.

Table 1 shows the summary of representative micromixers.

## 3. Droplet Generators

### 3.1. Classification and Working Principles

Microfluidic droplet generators are key components in microfluidic chips for generating highly homogeneous droplets, and they are widely used in the fields of material synthesis, drug screening, single-cell analysis, etc [91]. Precise control of droplet size, monodispersity, and generation frequency is critical for many applications. Some droplet generators utilize viscous shear to break droplets, with cross-flow, co-flow, and flow-focusing geometries being the three most common configurations [92,93,94]. Other methods enable droplet generation by altering channel geometry, such as step emulsification, microchannel emulsification, and membrane emulsification [95,96,97]. Depending on droplet generation mechanism, microfluidic droplet generators can be classified into the following groups, whose working principles are described in detail below. 

#### 3.1.1. Cross-Flow

**Figure 8:**
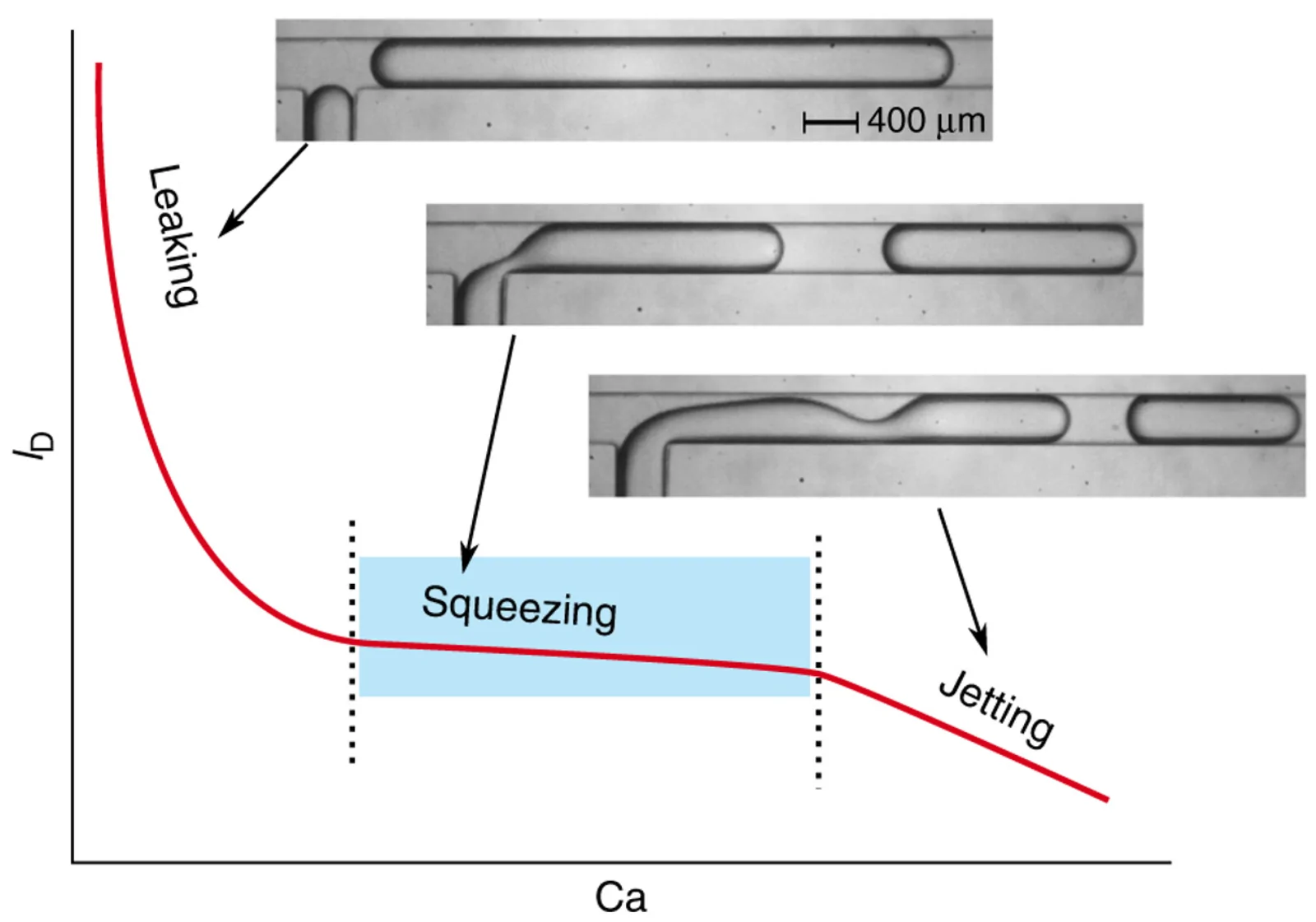
Schematic picture of the scaling of the normalized length of a droplet, with the Ca, as extracted from the full experimental data set, illustrating the leaking, squeezing, and jetting regimes. Reprinted with permission from Korczyk et al., Nat. Commun. 10, 9 (2019) [99]. Copyright 2019 Springer Nature.

The cross-flow consists of two microchannels perpendicular to each other. The dispersed-phase fluid is injected from the side channel perpendicularly to the main channel and meets the continuous-phase fluid flowing in the main channel at the channel intersection [98]. In cross-flow, the dispersed phase liquid is injected perpendicularly into a continuous phase fluid stream. Droplets are then detached from the dispersed phase nozzle tip under the influence of shear forces. This geometry is used extensively owing to its simplicity and ability to produce monodisperse droplets. T-shaped channel droplet size is influenced by the channel dimensions (especially the width of the continuous-phase channel), the two-phase flow rate ratio, fluid viscosity and interfacial tension. As shown in Figure 8, a researcher studied the leaking, squeezing, and jetting regimes at different capillary numbers (Ca). Lower Ca values usually correspond to the squeezing mode, in which the flow rate ratio of the two-phase flow determines the size of the generated droplets, while higher Ca values correspond to the dripping or jetting mode, in which the droplet size is mainly related to the continuous-phase fluid shear [99].

#### 3.1.2. Flow-Focusing

**Figure 9:**
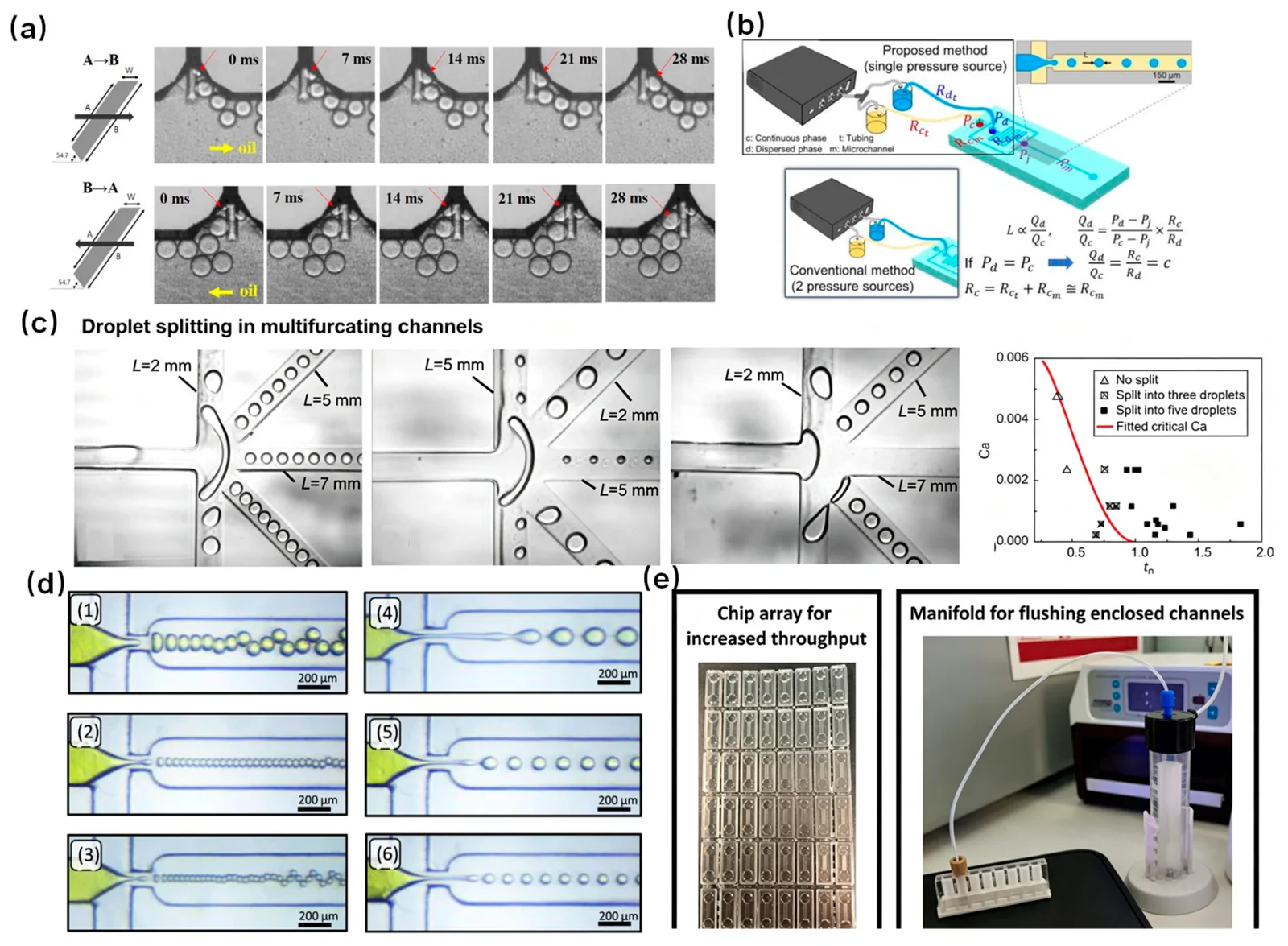
Droplet generators with different geometries. (**a**) Snapshots of water droplets. Reprinted from Cho et al., Micromachines 14, 223 (2023) [103], an open access article distributed under the terms of the Creative Commons CC BY license. Copyright 2023 by the authors. (**b**) Schematic diagrams of proposed and conventional methods of supplying fluids by flow-focusing devices. Reprinted with permission from Kalantarifard et al., Chem. Eng. Sci. 261, 8 (2022) [104]. Copyright 2022 Elsevier. (**c**) Schematic structure of a T-shaped droplet generator with weaving at channel crossings. Reprinted from Agnihotri et al., Phys. Fluids 37, 051304 (2025) [101], an open access article distributed under the terms of the Creative Commons CC BY license. Copyright 2025 by the authors. (**d**) Six distinct droplet generation regimes are achieved in a machine learning-automated designed microfluidic flow-focusing device. Reprinted by permission from Springer Nature: Lashkaripour et al., Nat. Commun. 12, 25 (2021) [105]. Copyright 2021. (**e**) The structure of a co-flow channel and the device fabricated by 3D printing. Reprinted from Desire et al., Anal. Chim. Acta 1384, 344971 (2026) [106]. Copyright 2026 Elsevier.

In a flow-focused droplet generator, a dispersed-phase fluid is injected from a central channel, surrounded on both sides by continuous-phase fluids that flow together through a narrow focusing hole. Here, the dispersed phase fluid is hydrodynamically compressed by two or more continuous phase fluid streams from opposing sides, converging it towards a narrow orifice. This focusing effect intensifies the shear forces, thereby promoting efficient droplet formation and enhancing their monodispersity. This geometry induces high shear stress and a pressure gradient concentrated at the narrow orifice. When the dispersed phase enters the channel intersection, the viscous shear forces from the continuous phase disrupt the balance between the interfacial tension and the dispersed phase, rendering it unstable and causing it to break into droplets [100]. Youngseo Cho et al. developed a microfluidic device with an asymmetric trapezoidal cross-section that, by reversing the continuous phase flow direction, enables the generation of two distinct sizes of monodisperse droplets from a single device (Figure 9a). As shown in Figure 9c, it demonstrates droplet splitting behaviors into 1, 3, or 5 daughter droplets in multifurcating microchannels with varied branch lengths, and quantifies the corresponding critical capillary number transition boundary via a phase diagram [101]. The size of flow-focused droplets is mainly influenced by the size of the focusing pore, the ratio of two-phase flow rates, fluid viscosity, and interfacial tension. Compared to T-shaped channels, flow focusing is more likely to go into jetting mode, generating smaller droplets, even down to the nanometer scale. The flow-focusing channels are typically used to generate spherical monodisperse droplets rather than plug-shaped droplets [102].

#### 3.1.3. Co-Flow

**Figure 10:**
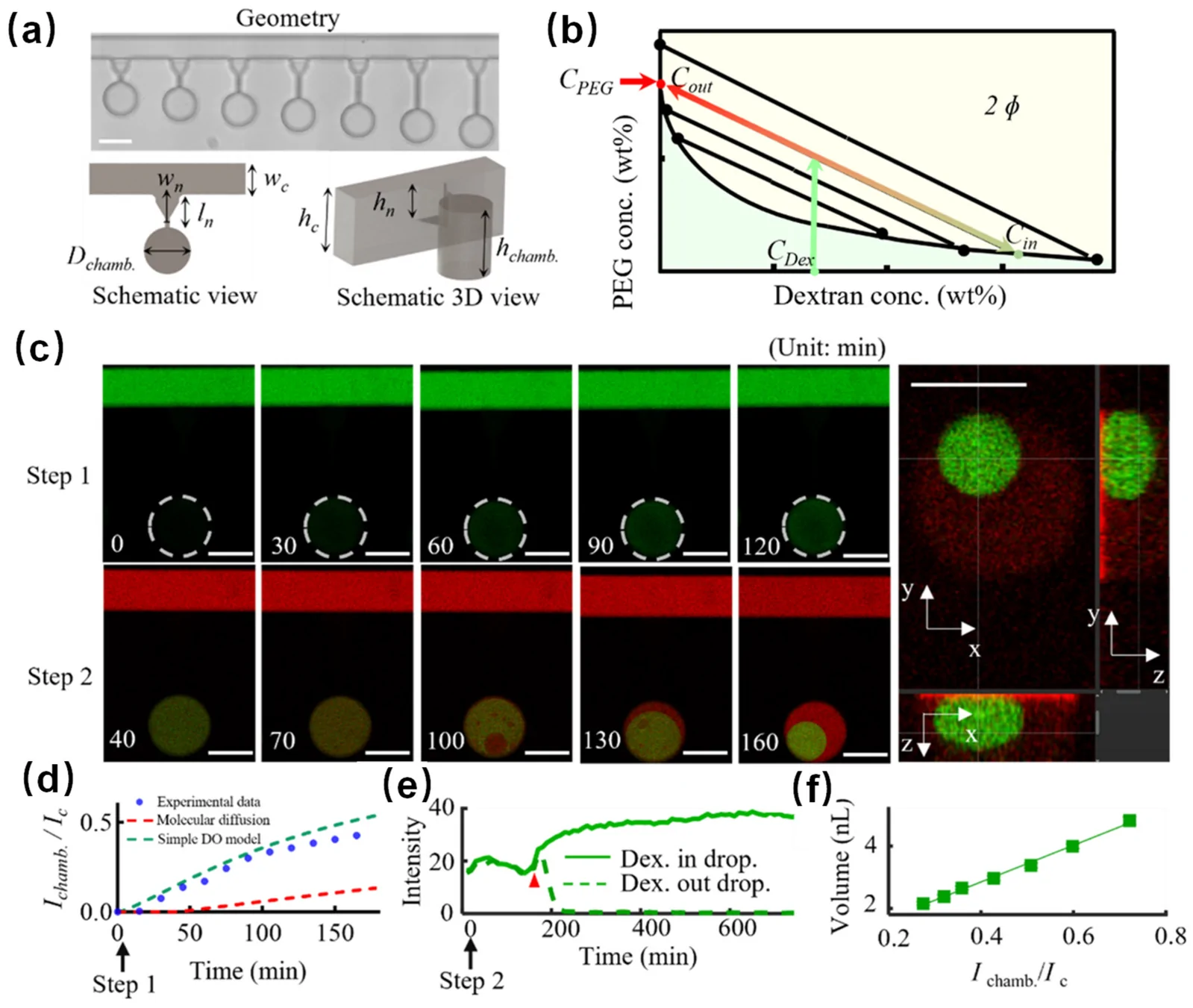
Microfluidic co-flow strategy for the controlled generation and trapping of membraneless all-aqueous droplets. Reprinted from Li et al., Lab Chip 26, 1037 (2026) [108]. Copyright 2026 Royal Society of Chemistry. (**a**) Microfluidic geometry schematic for droplet trapping. (**b**) Phase diagram of peg-dextran aqueous two-phase system. (**c**) Time-resolved confocal imaging of droplet generation and trapping. (**d**) Concentration evolution: experimental data vs. theoretical models. (**e**) Dynamic fluorescence intensity of dextran inside and outside droplets. (**f**) Quantitative correlation between droplet volume and normalized chamber concentration.

In co-flow geometry, the dispersed and continuous phase fluids meet in parallel streams. Shear forces at the end of the dispersed-phase channel control droplet formation, leading to droplet generation via drop or jet patterns. While droplets generated in drop mode are highly monodisperse, droplets generated in jet mode are polydisperse. The properties of the droplets generated by the co-flow microfluidic device are similar to those generated by the flow-focusing channel [107]. Desire et al. fabricated a novel device based on a co-flow mechanism using microscale 3D printing and applied it to the production of single and double emulsions (Figure 9e). Monodisperse water-in-oil and oil-in-water single emulsions could be produced in the same device with a coefficient of variation in less than 3% [106]. Figure 10 illustrates another interesting co-flow approach, where Li et al. developed a method for the controlled generation and trapping of membraneless water-in-water droplets [108]. In this system, the ultra-low interfacial tension of an aqueous two-phase system (ATPS) enables the formation of biocompatible all-aqueous droplets without the need for oil or surfactants. The figure shows the microfluidic geometry, the phase diagram of the PEG–dextran system, time-resolved confocal imaging of droplet generation and trapping, and quantitative correlation between droplet volume and chamber concentration (Figure 10a–f). This all-aqueous platform is particularly attractive for biological applications where oil-based systems may interfere with sensitive biomolecules.

#### 3.1.4. Step-Emulsification

**Figure 11:**
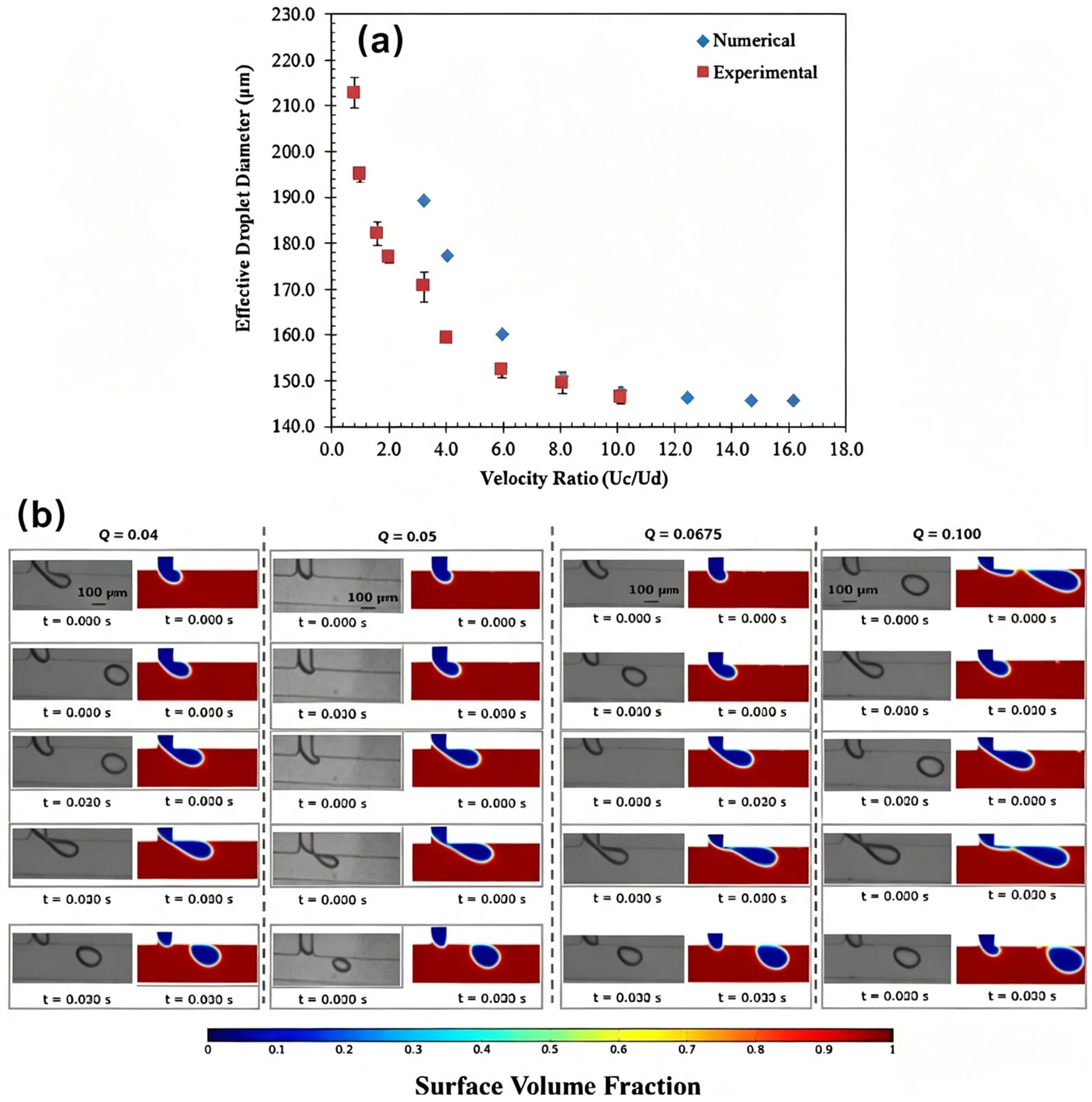
The process of droplet formation in the step-emulsification channel. (**a**) Effective droplet diameter between numerical and experimental result in the range of velocity ratio applied. (**b**) Droplet breakup phenomena in the range between Q of 0.04 and 0.1. Reprinted with permission from Wong et al., Chem. Eng. Res. Des. 144, 370–385 (2019) [111]. Copyright 2019 Elsevier.

Different from microfluidic devices that use shear to break the fluid to generate droplets, other geometries generate homogeneous droplets through variations in channel confinement where capillary pressure changes dramatically and droplets are squeezed due to interfacial tension (Figure 9d). Step emulsification utilizes sharp, geometric changes in channel height to generate droplets with remarkable monodispersity. The underlying physical mechanism is a Laplace pressure instability. As the dispersed phase filament emerges from the shallow microchannel into the deep reservoir, the change in curvature at the fluid front creates a pressure difference that drives the neck of the filament to collapse and pinch off a droplet [109]. Because this process is dominated by interfacial tension and geometry rather than flow rates, it is exceptionally robust against pressure fluctuations and allows for massive parallelization, enabling ultra-high-throughput production. A snapshot of the droplet formation process in the step-emulsion droplet generator obtained by the researcher is shown in Figure 11 [105]. Stepped emulsion geometries have high horizontal-to-vertical ratios and convert stable co-flows into droplets as they reach steep geometric steps confined by reservoir release channels. Based on the geometric characteristics of the steps, step emulsification can be categorized into horizontal and vertical steps. By adjusting the critical speed and parallelizing the droplet forming units, the yield of emulsification can be increased. These advantages drive the application of step emulsification technology for efficient large-scale production of monodisperse droplets [110].

#### 3.1.5. Active Droplet Generation

Active droplet generators are usually used in combination with passive methods to promote stable droplet generation by applying different external force fields [112]. Due to the presence of large resistance in the microchannels and the high compressibility of the fluid, the passive micromixer takes a longer time to stabilize the droplet generation. In addition, due to the complexity of the rheological properties, the fluid may exhibit anti-compression behavior during passive droplet generation [113]. Thus the fluid flow can be controlled by adding an external physical force field [114]. Active droplet generation offers more operational means and greater flexibility than passive droplet generation, allowing more precise control of droplet size and generation frequency. In some cases, on-demand droplet generation can be realized, whereas in passive droplet generation, it is difficult to control droplet size and generation frequency independently. In addition, active droplet generation systems respond much faster than passive methods, with response times reduced to milliseconds for active methods compared to seconds or even minutes for passive methods [29].

Surface acoustic wave (SAW) droplet generators leverage the radiative force produced by acoustic waves to facilitate droplet generation. These waves are typically induced on the surface of a piezoelectric substrate by fabricating interdigitated transducers and applying a high-frequency electrical signal. The resulting high-frequency acoustic vibrations on the substrate surface effectively shear the liquid into minute droplets, a mechanism often employed for the on-demand generation of extremely small and highly monodisperse droplets [115]. This SAW droplet generator enables droplet generation on demand, with precise control over droplet size and generation frequency [116]. The physical mechanism relies on the transfer of momentum from the high-frequency acoustic wave to the fluid, creating a force known as the acoustic radiation force. When a focused SAW beam is directed at the fluid–fluid interface, it induces a localized, high-pressure region. This force, when applied in a short pulse, provides the necessary energy to rapidly overcome surface tension and pinch off a single droplet from the static or slowly flowing dispersed phase [117]. The droplet volume can be precisely tuned by adjusting the applied power, while the generation timing is controlled down to the microsecond level by the electronic trigger signal. Within this particular flow focusing geometry and under the reported operating conditions, the SAW-based active control decouples droplet size from flow rate, offers a level of control that is not readily achieved by passive methods in the same configuration. We acknowledge that passive step emulsification and geometry-dominated regimes can also reduce flow rate sensitivity in their respective designs.

**Figure 12:**
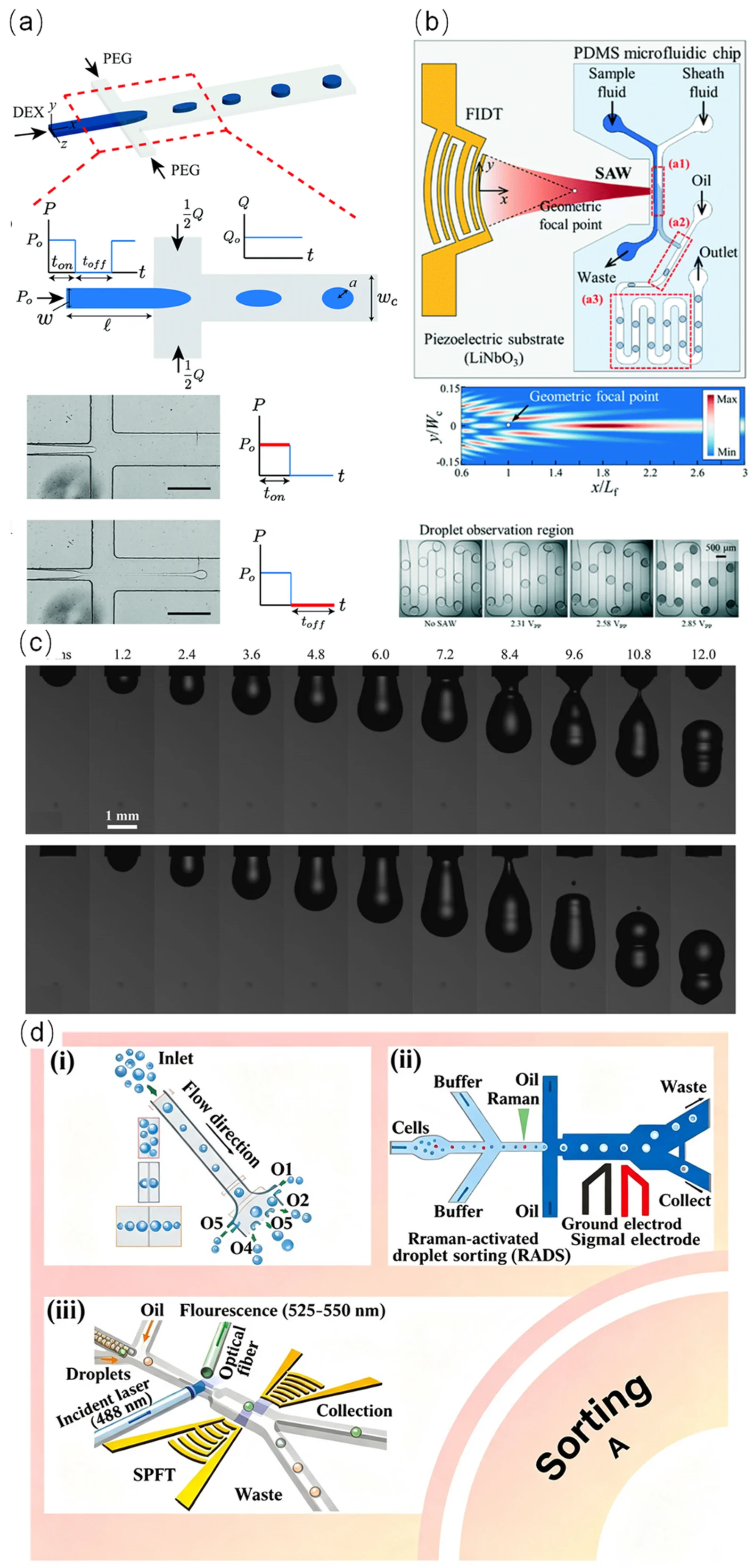
Examples of active and passive droplet operations. (**a**) Combining classical microfluidic flow focusing geometries with precisely controlled pulsating inlet pressures to form monodisperse aqueous two-phase system droplets. Adapted and used with permission from Moon et al., Lab Chip 15, 2437–2444 (2015) [118]. Copyright 2015 Royal Society of Chemistry, permission conveyed through Copyright Clearance Center, Inc. (**b**) Passive droplet generation in a T-junction followed by surface acoustic wave actuation for enhanced intra-droplet mixing, rather than active generation. Adapted and used with permission from Park et al., Lab Chip 20, 3922–3929 (2020) [119]. Copyright 2020 Royal Society of Chemistry, permission conveyed through Copyright Clearance Center, Inc. (**c**) High-speed sequential imaging of droplet formation dynamics in the valve-based on-demand droplet generator. Reprinted from Wang et al., AIP Advances 12, 095315 (2022) [120]. Copyright 2022 AIP Publishing LLC. (**d**) External field-assisted approaches for microfluidic droplet sorting. (**i**) Inertia-based sorting of hydrogel droplets. (**ii**) Raman-activated droplet sorting system. (**iii**) Acoustic chip with SPFT for droplet sorting. Reprinted with permission from Zhang et al., Research 8, 0856 (2025) [121]. Copyright 2025 American Association for the Advancement of Science.

As shown in Figure 12a, in the microfluidic flow focusing geometry, inlet pressure is precisely controlled to form monodisperse ATPS droplets. In this case, the dextran dispersed phase enters through a central inlet with a variable on–off pressure cycle controlled by a pneumatic solenoid valve [118]. In addition, coupling the acoustic waves to the airflow also allows the flow mixing of the aqueous solution to be controlled by forming acoustic flow-induced clusters of micronuclei. As shown in Figure 12b, the mixed stream is separated at the bifurcation, where a mixed stream with a precisely controlled chemical concentration is fed into a T-joint to produce droplets with adjustable chemical concentration [119]. It is important to distinguish between active droplet generation and other active operations performed on droplets. Figure 12b illustrates a case where droplets are generated passively in a T-junction, and surface acoustic wave actuation is subsequently used to enhance mixing within the droplets rather than to actively generate them. This example therefore represents passive droplet generation combined with active intra-droplet mixing. Figure 12c presents high-speed snapshots of droplet formation under two valve actuation modes, verifying the wide size-tunability of this on-demand droplet generator [120]. Similarly, Figure 12d depicts external field assisted approaches for droplet sorting, which is a downstream manipulation operation rather than an active generation method [121].

In addition to acoustic actuation, electrically driven approaches have significantly expanded the capabilities of active droplet microfluidics. Electrohydrodynamic (EHD) actuation leverages electric field-induced interfacial stresses to achieve on-demand droplet generation, transport, splitting, and merging, with millisecond-scale response times [122,123]. Charge injection and corona discharge methods, utilizing a needle-plate electrode to inject charges into a dielectric liquid layer, enable precise spatial and temporal control over droplet location, size, and quantity by modulating discharge voltage and duration [124]. Electrostatic manipulation on open surfaces, such as triboelectric-driven droplet motion on superhydrophobic substrates, has demonstrated velocities exceeding 100 mm·s^−1^ and accelerations above 10,000 mm·s^−2^, offering channel-free platforms ideal for digital microfluidics and point-of-care diagnostics [125,126]. These electrically driven techniques are intrinsically linked to the integration theme of this review, as they provide unique advantages for achieving precise temporal coordination between mixing and droplet generation, directly addressing the synchronization challenges discussed in Section 4.1.

### 3.2. Droplet Control

Droplet generation in microfluidic systems typically occurs at the confluence of two immiscible fluids within a microchannel. The breakup of the dispersed phase into discrete droplets at this junction is governed by the interplay between interfacial tension and shear forces, which are influenced by factors such as flow rate, channel geometry, and fluid properties (composition and viscosity) [127]. The generation of droplet sizes and frequency is directly related to the hydrodynamics. The dispersed and continuous phases also depend on the wettability of the channel walls [128]. For instance, a hydrophobic fluid will preferentially wet a hydrophobic surface [129]. Variations in surface properties along the channel length or over time can lead to droplet adhesion to the walls, causing issues such as undesirable transfer of contents between droplets, increased droplet size variability (polydispersity), and adsorption of analytes onto the channel surfaces. Therefore, the surface characteristics of the microchannel, which dictate fluid–wall interactions, are paramount for achieving stable and reliable droplet flow throughout the entire process, encompassing generation, merging, separation, storage, and analysis.

#### 3.2.1. Fluid Properties

The principle of droplet formation is to break the interfacial tension of the discrete phase by utilizing the flow shear stress of the continuous phase to split the discrete phase into nanoliter or even picoliter droplets. In particular, lower interfacial tension favors the formation of smaller droplets. During droplet generation, the viscosity of both the continuous phase and the dispersed phase affects the droplet size and generation frequency. In general, an increase in the viscosity of the continuous phase leads to an increase in droplet size, while an increase in the viscosity of the dispersed phase leads to decrease in droplet size [130]. Surfactants affect droplet formation and stability by reducing interfacial tension, preventing droplet aggregation, and altering the wettability of the channel surface [131]. In microdroplet generators, the two main fluid properties that affect droplet size and stability are fluid viscosity and interfacial tension. The capillary number, defined as Ca = μU/γ, where μ is the continuous phase viscosity, U is the characteristic flow velocity, and γ is the interfacial tension, represents the ratio of viscous forces to interfacial tension and determines the droplet formation regime. In the jetting mode, droplet size decreases with increasing Ca; in the leaking mode, droplet size is highly sensitive to viscosity at low Ca. The viscosity of the droplet phase has a small effect on droplet formation at low Ca, but the high viscosity contrast (μd/μc) may introduce additional flow resistance that needs to be considered in the modeling [99].

#### 3.2.2. Flow Rate

The flow rate ratio is an important parameter to control the droplet size and generation frequency. In squeezing mode, droplet size is mainly determined by the flow rate ratio. In the dripping mode, the droplet size decreases with the increase in the continuous phase flow rate [132]. In jetting mode, droplet size is proportional to the square root of the flow rate ratio. The Ca, defined as the ratio of the viscous force to the interfacial tension, reflects the competition between the viscous force and the interfacial tension and affects the droplet formation mode. In the squeezing mode, the effect of Ca is negligible. In the dripping mode, the droplet size is proportional to the inverse of Ca. A low Ca number (Ca << 1) indicates that interfacial tension dominates, droplets do not deform easily and form large, stable droplets. A high Ca number (Ca >> 1) indicates that viscous forces dominate, and the droplet is easily deformed and broken, resulting in a small and unstable droplet [133]. In addition, an increase in the continuous phase flow rate enhances the shear force and promotes droplet fragmentation and deformation to form smaller droplets [99]. In contrast, an increase in the dispersed phase flow rate increases the droplet generation frequency and size of droplet generation.

#### 3.2.3. Channel Dimensions

When the channel size is much larger than the droplet size, the channel size has less effect. When the channel size is comparable to the droplet size, the channel size restricts the growth of the droplets and affects the shape of the droplets. Therefore, in microfluidic channels, the channel size can control the generation of highly homogeneous droplets. Channel width (w) and height (h) affect the size and shape of the droplet. Smaller channel dimensions usually result in smaller droplets. Channel aspect ratio (h/w) affects droplet stability and shape. The wettability of the channel surface determines which fluid acts as the continuous phase, as the continuous phase should preferentially wet the channel wall to ensure stable droplet generation and prevent droplet adhesion [134]. Therefore, hydrophobic (or oleophilic) channel surfaces are typically employed for the generation of water-in-oil (W/O) droplets, as the oil phase preferentially wets the channel wall. Conversely, hydrophilic (or oleophobic) surfaces are typically used for the generation of oil-in-water (O/W) droplets, where the aqueous phase acts as the continuous phase [135]. The geometry of intersections (e.g., T-intersections, fluid-focused intersections, coaxial intersections) affects the droplet formation pattern and size. The height and length of steps in a step emulsion structure affect droplet size and monodispersity.

**Figure 13:**
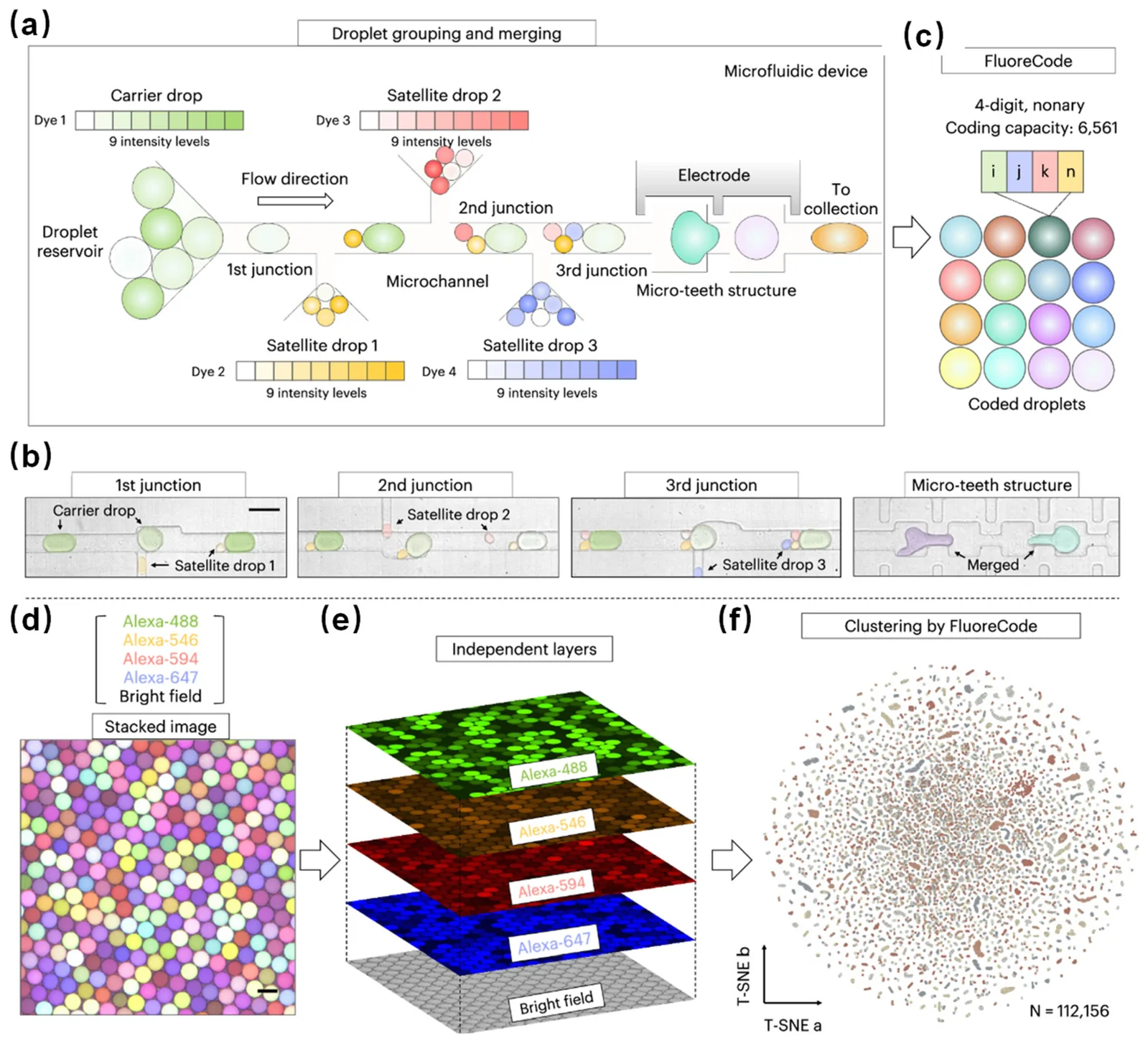
Artificial intelligence-accelerated high-throughput screening of antibiotic combinations on a microfluidic combinatorial droplet system. (**a**) DropAI generates massive combinations in droplet reactors with a multi-droplet microfluidic merger. (**b**) Micrographs depict the droplet pairing and merging process. (**c**) As every droplet is coded by fluorescent color and intensity, the merged droplets are rendered a 4-color barcode (FluoreCode) indicating the exact combinations. (**d**) A stacked micrograph displaying the as-built combinatorial pool. (**e**) For decoding, the merged droplets are imaged under 4 fluorescent channels to extract the FluoreCode of each droplet. (**f**) A t-distributed stochastic neighbor embedding (T-SNE) projection of 153302 droplets containing 6527 recognized clusters, ~99.5% of the entire combinatorial space. Reprinted from Zhu et al., Nat. Commun. 16, 2720 (2025) [137]. Copyright 2025 Springer Nature.

In summary, the generated droplets depend on the balance between fluid characteristics, channel structure design, and flow velocity. Although the fundamental principles of droplet generators are well understood, the current research frontier lies in optimizing their performance and robustness. To this end, researchers are increasingly leveraging machine learning technologies, not only to refine operational control through real-time feedback loops but also to design generator geometries from scratch that maximize stability and monodispersity [136]. Some scholars have integrated object detection models based on convolutional neural networks, decision algorithms, and feedback control algorithms into the system to achieve automation of droplet generation and collection. In addition, an accelerated high-throughput combination drug evaluation system has been achieved through artificial intelligence. As shown in Figure 13, this system can not only generate high-throughput combinations of antibiotics at different concentrations, but also automatically analyze the effects of different antibiotic combinations on the dynamic growth of bacteria [137]. This intelligent approach to design and operation marks a significant step toward achieving fully automated and reliable droplet-based platforms.

### 3.3. Performance Characterization

#### 3.3.1. Droplet Size Distribution

The droplet size distribution describes the proportion of droplets of different sizes in the population. Ideally, the size distribution of microfluidic droplets should be as narrow as possible, i.e., well monodisperse, to ensure consistent experimental results. The polydispersity is usually quantified by the coefficient of variation, defined as the ratio of the standard deviation of the droplet radius to the mean radius. The smaller the coefficient of variation (CV), the better the monodispersity of the droplets.

Dynamic light scattering (DLS) is a common method for characterizing the size distribution of a droplet, DLS determines the hydrodynamic diameter of a droplet by measuring the fluctuations in the intensity of scattered light caused by the Brownian motion of the droplet [138]. For non-spherical droplets, DLS measures the diameter of a sphere with the same diffusion coefficient as the droplet. In addition, Nanoparticle tracking analysis (NTA) can be used to measure droplet size distribution and concentration. NTA determines droplet size by tracking the Brownian motion of individual droplets and analyzing their trajectories. Microscopic imaging combined with image analysis software can be used to directly measure droplet size and statistically analyze the droplet size distribution [139]. Transmission electron microscopy provides higher-resolution images of droplets and can be used to measure droplet size and morphology.

A study demonstrated a novel approach for generating highly monodisperse droplets in microfluidic devices by applying uniform pressure from a single source [104]. Unlike conventional methods, this approach isolates droplet generation from external fluctuations arising from imperfections in the flow source. The generality of this method was validated through its successful implementation in both flow-focusing and co-flow devices, commonly used for high-frequency droplet production. A comprehensive analytical model, incorporating all relevant system dynamics and both internal and external factors affecting monodispersity, was developed. This model characterizes the dynamics of segmented two-phase flow by drawing an analogy to single-phase flow, akin to electron transport in an electrical circuit. Based on this analysis, design principles were established to facilitate the generation of hypermonodisperse droplets. These guidelines focus on minimizing pressure discontinuities at the droplet-forming junction. This is achieved by reducing the pressure difference between the dispersed and continuous phases and lowering interfacial tension, thereby effectively approximating the behavior of a single-phase system. Additionally, diminishing the hydrodynamic resistance within the main channel was identified as essential for enhancing monodispersity. The implementation of these recommendations regarding flow source configuration and channel network architecture successfully mitigated both internal and external fluctuations, leading to a droplet CV below 0.2% [140].

#### 3.3.2. Generation Frequency

Droplet generation frequency is the number of droplets generated per unit time. High-throughput applications require a high frequency of droplet generation. Droplet generation frequency can be determined by using a high-speed camera to record video of droplet generation and then analyzing the number of video frames [141]. However, the disadvantage of this method is that it is costly and requires a high-speed camera and image processing software. Alternatively, the droplet generation frequency can be calculated from the dispersed phase flow rate (*Q_d_*) and droplet volume (*V*).

(2) $$f=\frac{Q_{d}}{V}$$

Similarly to the droplet size distribution, the droplet generation frequency is also affected by fluid properties, flow rate, channel geometry and external fields.

#### 3.3.3. Stability

Droplet stability is the ability of a droplet to resist aggregation, breakup, or adhesion to the channel wall. Stable droplets are critical for many applications, such as long-term cell culture, drug delivery, and material synthesis. Common methods for assessing droplet stability include observing the flow behavior of a droplet in a microfluidic device and monitoring changes in droplet size and morphology over time [142]. Droplet stability can be quantified by measuring the time of aggregation or breakup, and zeta potential can be used to assess the electrostatic stability of a droplet. Droplet stability is influenced by several factors including fluid properties (interfacial tension, viscosity, viscosity ratio), surfactant type and concentration, channel surface wettability, flow rate, and external fields. Characterization of the performance of microfluidic droplets is critical to assess their suitability for a variety of applications. By accurately controlling and measuring droplet size distribution, generation frequency, and stability, the design and operating parameters of microfluidic devices can be optimized for different applications [143].

Table 2 shows the ummary of representative droplet generators.

## 4. Integration and Synergistic Applications

The integration of micromixers and droplet generators into microfluidic chips enables precise control of reaction conditions within individual droplets, facilitating high-throughput experiments and providing isolated microenvironments for a wide range of chemical and biological processes, which offers further potential applications. In this section, the focus is on integrated devices, including their design, applications, materials, and processing methods. 

### 4.1. Advanced Integration Strategies for High-Performance Integrated Devices

**Figure 14:**
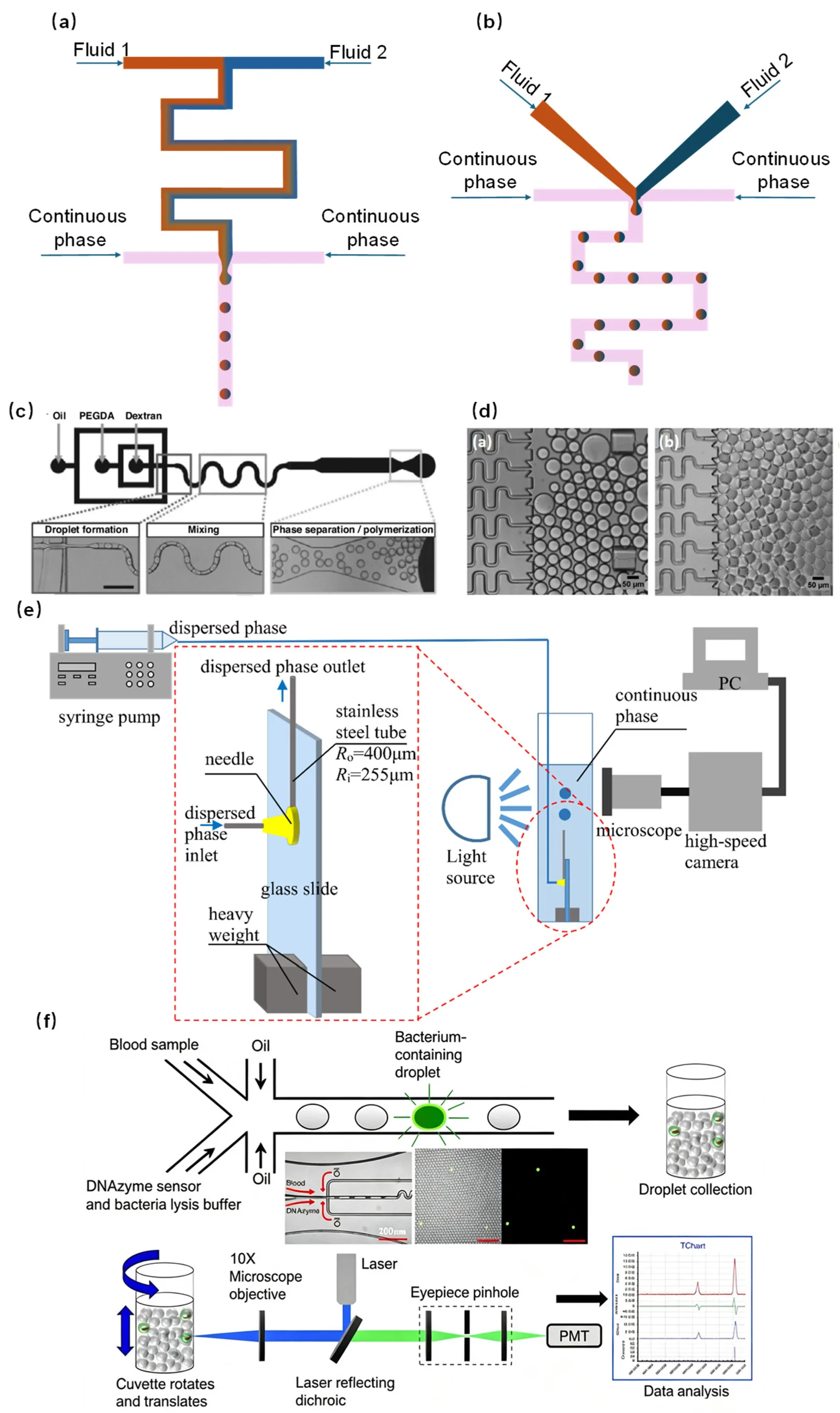
Two strategies of integration devices and devices of mixing in droplets. (**a**) Mix-then-encapsulate. (**b**) Encapsulate-then-mix. (**c**) Droplet microfluidics-based biomedical microcarriers. Reprinted with permission from Ma et al., Microsyst. Nanoeng. 12, 53 (2026) [146]. Copyright 2026 Springer Nature. (**d**) Control and detection of chemical reactions in microfluidic systems. Reprinted with permission from Chung et al., Micromachines 10, 592 (2019) [145]. Copyright 2019 by the authors. (**e**) Pictorial representation of the experimental setup showing a computer used for droplet data recording. Reprinted with permission from Shen et al., Micromachines 11, 962 (2020) [147]. Copyright 2020 by the authors. (**f**) The design of the micro-mixers and the water droplets with different trypan blue concentrations generated in individual microchannels. Reprinted from Nan et al., Curr. Opin. Chem. Eng. 52, 101262 (2026) [148], an open access article under the CC BY license. Copyright 2026 by the authors.

The integration of micromixers and droplet generators is predicated on a fundamental architectural choice: whether to mix reagents before or after they are partitioned into droplets. As shown in Figure 14, this decision gives rise to two primary strategies: “mix-then-encapsulate” and “encapsulate-then-mix” [144,145]. The optimal choice depends on the specific requirements of the application, such as the need for reaction homogeneity, precise temporal control, and system simplicity. Table 3 shows the comparison of integration strategies: mix-then-encapsulate vs. encapsulate-then-mix.

The two integration strategies differ fundamentally in reaction start time, mixing time and compositional variation, premature reaction, droplet stability, fabrication complexity, and suitability. For reaction start time, mix then encapsulate initiates the reaction immediately after mixing before droplet formation, whereas encapsulate then mix allows triggering at a controlled later time after droplet generation and merging. Regarding mixing time and compositional variation, upstream mixing achieves bulk homogenization within the micromixer, yielding highly uniform composition though not absolutely identical due to flow fluctuations and segmentation, while intra-droplet mixing occurs after encapsulation and its efficiency depends on internal circulation and channel geometry, potentially introducing slight compositional gradients. Premature reaction is unavoidable in upstream mixing, which can be detrimental for fast kinetics or unstable reagents, while the encapsulate then mix avoids this by keeping reagents separate until the desired moment. Droplet stability is unaffected by upstream mixing, but intra-droplet mixing with bends or active elements may lead to deformation or coalescence. Fabrication complexity is lower for mix then encapsulate, requiring only a passive micromixer, whereas the encapsulate then mix demands more complex geometries such as serpentine channels or integrated electrodes. Suitability also differs: upstream mixing suits slow reactions and quantitative assays like digital PCR where uniformity is critical, while intra-droplet mixing is preferred for fast reactions, single-cell analysis, and sequential multi-step processes requiring precise temporal control. As shown in Table 1, we present a comparison of two integration strategies: mix-then-encapsulate and encapsulate-then-mix.

#### 4.1.1. Mix-Then-Encapsulate

The straightforward strategy is to place a micromixer upstream of the droplet generation unit (Figure 14a). In the “mix-then-encapsulate”, two or more fluids containing reagents or cells are homogenized before they are introduced into the dispersed phase inlet of the droplet generator. Passive mixers, such as serpentine or herringbone, are commonly used to ensure that the fluid entering the droplet nozzle is a homogenous mixture. The primary design goal is to achieve mixing in the residence time before encapsulation, ensuring that every droplet formed has a highly uniform chemical composition. As shown in Figure 14e, this study investigates droplet generation and encapsulation in flow-focusing droplet generators, showcasing droplet microfluidics as microreactors. While dedicated integrated designs of ‘micromixers’ and ‘microdroplet generators’ are not extensively detailed, existing applications such as in-droplet reactions and microparticle encapsulation exemplify the integration of droplet generation with subsequent in-droplet operations (e.g., mixing, reaction, encapsulation) on a single microfluidic platform. Sangam et al. developed a system that integrates micro-mixers to create a concentration gradient by uniformly mixing two aqueous solutions (Figure 14f). This mixed solution then flows into on-chip flow-focusing devices, which generate droplets. This design demonstrates a microfluidic platform where mixing and droplet formation are intrinsically linked and performed sequentially.

This method is applied to achieve high homogeneity within each droplet. It is essential for quantitative assays such as droplet digital PCR (ddPCR), where the PCR master mix must be perfectly uniform before being partitioned with the DNA sample to ensure reliable amplification in every droplet [149]. The architecture is relatively simple to design and fabricate. Since the mixing is completed before droplet formation, the downstream droplet generation is not complicated by additional active components or complex channel geometries.

The primary drawback of upstream mixing is the lack of precise temporal control. Once the reagents are mixed, the reaction begins immediately. This is problematic for studying rapid reaction kinetics or for processes where reactions should only be initiated after encapsulation. In applications such as single-cell analysis, pre-mixing a cell suspension with a lysis buffer would lyse all cells in the bulk phase before they could be isolated into individual droplets. This makes the upstream mixing approach unsuitable for applications requiring the compartmentalization of discrete entities before a chemical treatment.

Residence time, the duration reagents spend in the micromixer before reaching the droplet generator, is critical. A key design challenge is that residence time cannot be independently tuned from droplet generation frequency, as both depend on the same flow rates. For fast reactions, the residence time also determines how much reaction has occurred before encapsulation.

#### 4.1.2. Encapsulate-Then-Mix

In contrast, the “encapsulate-then-mix” strategy involves initiating mixing after the droplets have been formed (Figure 14b). This is achieved either by generating droplets that contain initially unmixed reagents or by merging two different droplet populations downstream. After generation, droplets are passed through winding or serpentine channels. The curved path creates internal secondary flows within the moving droplet, which fold and stretch the internal fluid interfaces to accelerate mixing. As shown in Figure 14c, integrated devices are used in cell culture for encapsulating cells and providing a homogeneous mixture of nutrients and growth factors, thereby promoting cell proliferation and differentiation. In drug screening, these devices enable high-throughput generation of microdroplets containing diverse drug combinations, enabling rapid mixing and reaction initiation. Furthermore, integrated devices enable the development of automated and miniaturized experimental platforms, reducing reagent consumption and enhancing experimental efficiency and reproducibility. Additionally, Figure 14d illustrates another approach where droplets are initially generated, followed by accelerated fluid mixing within the droplets as they traverse a serpentine channel.

The “encapsulate-then-mix” approach allows a reaction to be triggered at a precise time and location on the chip, making it ideal for studying reaction kinetics, performing sequential chemical steps, and ensuring that a specific action occurs only after a cell has been isolated. This strategy is essential for single-cell genomics and proteomics. A single cell can be encapsulated first, and then a second droplet containing lysis buffer and reagents can be merged with it, ensuring that the molecular contents of that specific cell are analyzed without cross-contamination [150]. Passive intra-droplet mixing is often slower than bulk mixing due to the low Reynolds number within the droplet, potentially becoming the rate-limiting step of the entire process. Implementing active mixing components or reliable droplet merging junctions increases the complexity of device fabrication and operation, requiring integrated electrodes, transducers, or precisely engineered channel geometries. The forces applied for active mixing or merging can, if not carefully controlled, lead to droplet instability or unintended coalescence.

The relevant timescale is the intra-droplet mixing time. In serpentine channels, each turn induces internal recirculation that reduces mixing time. The total channel length after generation must provide sufficient residence time (i.e., number of turns) to achieve the required mixing before droplets reach the outlet.

In summary, the choice between these two fundamental strategies represents a critical trade-off between simplicity/homogeneity (upstream mixing) and temporal control/reaction isolation (intra-droplet mixing). The continued innovation in both these areas is driving the development of increasingly sophisticated and powerful integrated microfluidic platforms for a wide range of applications.

#### 4.1.3. Engineering Considerations and Practical Challenges

In integrated devices, pressure drops of the micromixer and droplet generator must be balanced. Excessive mixer resistance can induce pulsatile flow, pressure fluctuations that destabilize droplet formation, or device failure if burst pressure is exceeded. Thus, the mixer’s hydraulic resistance should ideally be comparable to or lower than that of the generator. In mixing after encapsulation, back pressure from downstream channels can shift the droplet generation regime or induce breakup. As a guideline, the mixing section pressure drop should not exceed 10–20% of the total system pressure.

Synchronization is also critical. In upstream mixing, the residence time between mixing and encapsulation must align with droplet generation frequency; flow fluctuations can cause droplets to encapsulate fluid mixed at unintended times, introducing population variability. In within-droplet mixing, actuation signals must be precisely timed with droplet arrival, achievable via optical detection or passive synchronization through tuned flow rates. Asynchronous operation in applications like single-cell lysis leads to empty droplets, multiple cells, or incomplete mixing.

Cross-contamination risks differ. Upstream bulk mixing distributes any contamination across all droplets, particularly problematic in PDMS due to hydrophobic adsorption. Sequential runs require thorough washing, challenging given the high surface-to-volume ratio and complex geometries. Droplet compartmentalization in the encapsulate-then-mix strategy significantly reduces bulk contamination, though risks remain from unintended coalescence, surfactant failure, or leakage during merging. Surface passivation and inert materials are recommended.

Compatibility spans multiple aspects. Fluidically, passive mixing often requires Re > 10, while stable droplet generation operates at Ca < 0.1—flow rates optimizing one may compromise the other. Surfactants for droplet stability can alter micromixer wetting, while reagents like proteins may interfere with surfactant function. Operationally, throughput and mixing requirements may conflict. Thermally, gradients for assays like PCR can induce Marangoni effects. Materially, hydrophilic surfaces for protein-resistant mixing conflict with hydrophobic surfaces required for water-in-oil pinch-off, demanding spatially selective surface modification, a significant fabrication challenge.

### 4.2. Materials and Fabrication

The choice of materials and manufacturing methods for micromixers has a significant impact on their performance, cost and range of applications. Ideal materials for micromixers should have good processability, biocompatibility (for biomedical applications), chemical inertness (for chemical reaction applications), optical transparency (for optical detection applications), and suitable mechanical properties. Commonly used materials for micromixers include PDMS, glass, and thermoplastics. Various micromachining techniques are also widely used in the fabrication of micromixers. This section summarizes the materials and manufacturing techniques required for microfluidic devices that produce uniform droplets.

#### 4.2.1. Materials

PDMS has dominated academic research because it is easy to fabricate by soft lithography [151]. The process typically involves making a master mold on a silicon wafer using photolithography and then pouring PDMS onto the master mold. The cured PDMS can be easily peeled off, bonded to glass or another PDMS layer, and rendered hydrophobic through surface treatments such as salinization with hydrophobic silanes or by applying hydrophobic coatings. This process allows for rapid prototyping and cost-effective production of small batches of devices [152,153]. PDMS is optically transparent, biocompatible, and gas-permeable, making it suitable for cell culture and other biological applications. However, disadvantages of PDMS include its tendency to absorb hydrophobic molecules, which can lead to contamination and changes in droplet composition over time. It also swells in the presence of certain organic solvents, limiting its compatibility with some chemical assays. Moreover, the relatively low Young’s modulus of PDMS causes the channel to deform at higher pressures, thus limiting flow rates and throughput. While surface modifications can tailor the wettability of PDMS for a specific application, these modifications are typically not permanent and degrade over time, requiring careful consideration for long-term experiments [154].

Commonly used thermoplastics include polymethylmethacrylate (PMMA), polycarbonate (PC), cyclic olefin copolymer (COC), and others. Different types of thermoplastics have different properties, e.g., PMMA exhibits good optical transparency, and COC shows low water absorption. Thermoplastics offer a diverse range of materials for droplet microfluidics, each with its advantages and disadvantages. PMMA is a popular choice due to its good optical properties, ease of machining, and relatively low cost. However, it is less chemically resistant than glass or some other thermoplastics such as COC. Fluoropolymers offer exceptional chemical inertness and inherently superhydrophobic surfaces, making them ideal for water-in-oil droplet generation without the need for surface modifications [134]. However, fluoropolymers are more challenging to process due to their high melting points and chemical inertness, often requiring specialized bonding techniques. COC is a more recently adopted thermoplastic offering low water absorption, good chemical resistance, and compatibility with various solvents. The increasing availability of biocompatible thermoplastics has expanded their use in droplet-based biological assays. Thermoplastics are a class of plastics that can be repeatedly heated to soften and cooled to harden, offering advantages such as good processability, low cost and a wide range of material options.

Glass is a rigid material with excellent optical clarity, chemical inertness, and resistance to high temperatures and pressures, making it suitable for applications that require optical inspection or involve caustic chemicals. The main disadvantage of glass is that it is difficult and relatively expensive to work with. Common glass materials include quartz glass and borosilicate glass. In addition to these commonly used materials, several other materials are used in the fabrication of micromixers, such as ceramics, metals, and paper.

#### 4.2.2. Micromachining Techniques

**Figure 15:**
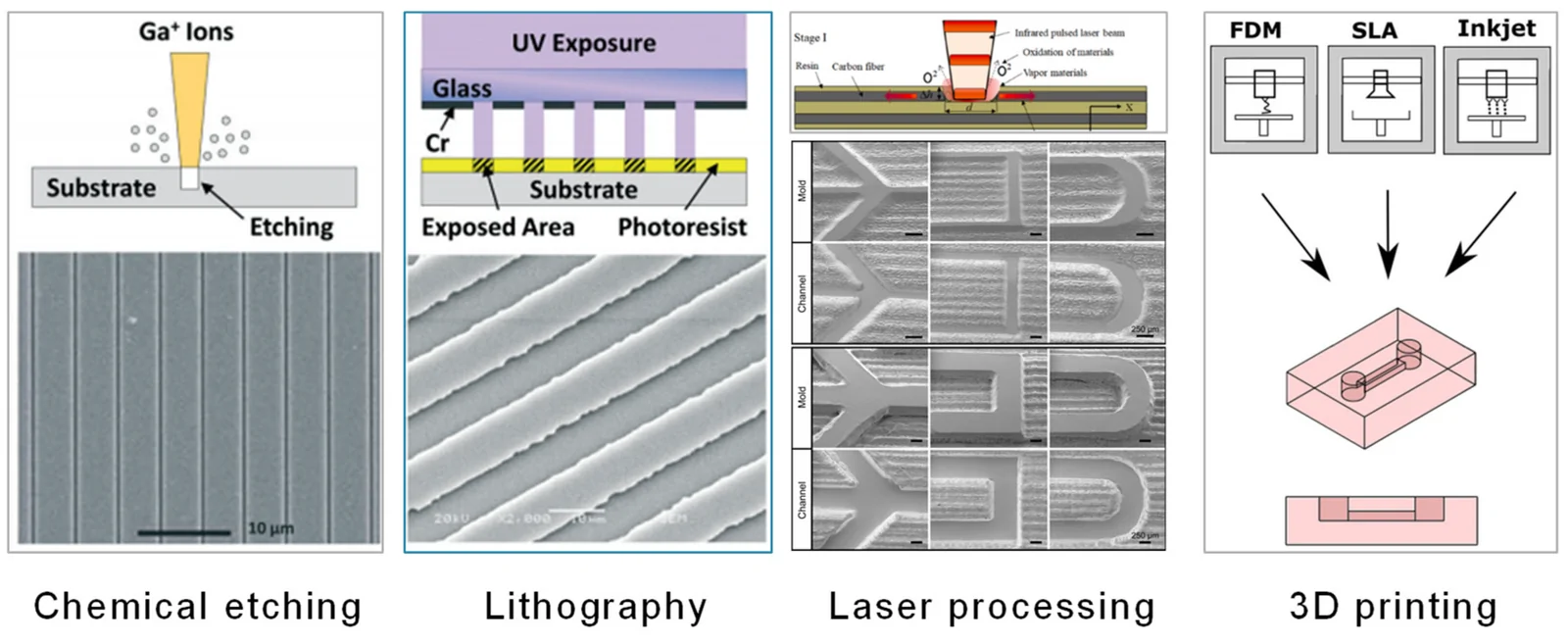
The common micro- and nano-channel processing technologies, including lithography, 3D printing, chemical etching, and laser processing. Adapted and used with permission from Hong et al., Lab Chip 16, 4296–4312 (2016) [155]. Copyright 2016 Royal Society of Chemistry, permission conveyed through Copyright Clearance Center, Inc. Reprinted with permission from Mehta et al., Bio-Des. Manuf. 4, 311–343 (2021) [156]. Copyright 2021 Elsevier [157].

The realization of sophisticated microfluidic devices, from simple channels to complex, multi-functional platforms, fundamentally depends on the capabilities of micromachining technologies. This choice of fabrication method becomes paramount for integrated systems, which demand the monolithic fabrication of heterogeneous functional units on a single substrate. The ability to create, align, and bond these different components not only dictates the device’s performance but also its robustness and scalability. This section provides an overview of key fabrication techniques, highlighting their respective strengths and limitations in creating these advanced integrated architectures (Figure 15).

Recent studies have systematically examined the critical role of post-processing and hybrid fabrication techniques in determining the practical performance of 3D-printed micromixers. Pricci and Percoco investigated the effect of ironing process parameters on material extrusion printed Y-micromixers and microfluidic gradient generators, demonstrating that optimized ironing line spacing and speed significantly enhance surface smoothness, leading to a 191% increase in mixing length at 10 µL/min and 198% at 20 µL/min compared to non-ironed devices, while also achieving stable concentration gradients in the crosswise flow direction [158]. Carnero et al. developed a versatile hybrid technique combining stereolithographic 3D printing with pulsed laser ablation to fabricate passive straight micromixers, and used computational fluid dynamics to elucidate the physical mechanisms of mixing enhancement, showing that this optical approach holds promise for manufacturing high-performance microfluidic mixing devices [159]. Wang et al. reported a directly moldable 3D PDMS micromixer based on a splitting–stretching–recombination design, fabricated via two-photon polymerization printing and soft lithography, which achieves a mixing efficiency above 0.90 for low Reynolds number solutions (0.01–10) with a mixing volume smaller than 20 nL, while overcoming the integration and assembly limitations of conventional 3D micromixers [160]. These studies collectively underscore that careful attention to surface finishing, hybrid fabrication strategies, and post-processing is essential for translating 3D-printed micromixers into reliable and high-performance devices.

Soft lithography is a commonly used PDMS microfluidic chip fabrication technique, which uses photolithography to create a master mold, and then the PDMS is poured onto the master mold and solidified [161]. Soft lithography is widely used for rapid prototyping due to its advantages of low cost, simple operation and mass production. In droplet microfluidics, soft lithography can fabricate precise microchannel structures with high resolution, such as T-junctions and flow-focusing channels, which are crucial for droplet formation. However, the elasticity of PDMS channels may lead to slight channel expansion during high-pressure or long-term droplet generation, affecting the uniformity of droplet size. Therefore, when designing high-precision droplet generators, channel wall thickness and geometry need to be considered to minimize the impact of elastic deformation.

Photolithography is a high-precision micromachining technology that utilizes photoresists and mask plates to fabricate microstructures on substrate materials through steps such as optical exposure and development. Photolithography can produce high-resolution microchannels and microstructures, but at a relatively high cost. However, its high precision is crucial for achieving sub-micron droplets or highly parallel droplet generators, which require extremely precise control over channel dimensions. When integrating droplet generation and detection modules, photolithography ensures precise alignment of optical paths and droplet channels.

Laser micromachining is a micromachining technology that utilizes a laser beam for material removal or modification, including laser ablation, laser cutting, and laser direct writing. Laser micromachining technology can process a variety of materials with the advantages of high precision, high efficiency and non-contact processing, etc. A consumer laser cutter was used to fabricate a microfluidic device for droplet formation in a study. The continuous and dispersed phases used for droplet generation were an oil phase, suitable for droplet digital polymerase chain reaction (PCR), and phosphate-buffered saline or a polyethylene glycol solution [162]. The advantage of laser micromachining lies in its flexibility and rapid iteration capability, allowing direct etching of the required geometric structures for droplet generation onto the substrate, making it particularly suitable for small-batch customization or rapid prototyping. However, the surface roughness of laser etching may be higher than that of photolithography, which requires additional consideration in certain droplet generation applications with extremely high demands for surface smoothness, to prevent droplet adhesion or rupture.

Three-dimensional printing technology can rapidly fabricate complex three-dimensional micromixer structures, such as Three-dimensional printing technology based on two-photon polymerization [163]. Three-dimensional printing technology has the advantages of design flexibility and rapid prototyping, but there are still some limitations in its resolution and material selection. For example, LCD 3D printing technology can fabricate micro hybrids with high transparency and complex structures. 3D printing offers unprecedented freedom in designing three-dimensional channels for droplet microfluidics, allowing the creation of structures like vertical flow-focusing or multi-layer complex mixing channels, which are difficult to achieve with traditional 2D fabrication. This holds immense potential for improving mixing efficiency, enabling multi-phase droplet generation, or constructing more complex functional droplet chips. The challenge lies in further increasing printing resolution to meet the demands for micron or even sub-micron droplet generation, and in developing more 3D printing materials with biocompatibility or special surface properties. When designing, it is crucial to fully leverage its three-dimensional advantages and consider how the three-dimensional geometry of the channels affects fluid dynamics and droplet formation mechanisms.

In addition to lithography-based and 3D-printed devices, glass capillary microfluidics represents an important complementary fabrication approach, particularly for co-flow droplet generators and rapid prototyping [163,164]. These devices are assembled from concentric cylindrical capillaries, typically a round inner capillary inserted into a square or round outer capillary, enabling truly axisymmetric co-flow and flow-focusing geometries that are difficult to achieve with planar lithographic techniques [163]. Glass capillary devices offer excellent chemical resistance, optical transparency, and the ability to handle a wide range of solvents without swelling or adsorption issues [164]. Their modular and reconfigurable nature allows for easy adjustment of capillary alignment and tip separation, facilitating the generation of single, double, and multiple emulsions with precise control over droplet size and morphology [163,165]. Recent developments include Lego-inspired block-based assemblies that reduce setup time from ~30 min to just several minutes, as well as rapid prototyping methods using pipette tips and commercially available capillaries [165,166]. While glass capillary devices are less amenable to high-throughput parallelization compared to photolithographically defined chips, they remain invaluable for applications requiring extreme chemical inertness, optical access, or complex multi-phase emulsion systems [167].

Chemical etching involves selective removal of material from a substrate using chemical etchants. Chemical etching can be used to create microchannels and other features in various materials, including glass, silicon, and metals. It is often used in conjunction with photolithography to create high-resolution patterns. One researcher has utilized friction-induced selective etching for the processing of micro- and nano-channels, which has been successfully applied to the preparation of micro-mixers and micro-droplet generators [12,13,166]. Micromechanical machining utilizes miniature cutting tools to remove material from a substrate, creating microfluidic channels and other features. Micromilling is often used for prototyping thermoplastic devices due to its relatively simple implementation and low cost. However, it offers lower resolution compared to lithographic techniques. Chemical etching can achieve highly smooth and chemically inert channel surfaces on glass and silicon substrates, which is crucial for preventing droplet contamination and maintaining stable surface wettability. Micromilling, on the other hand, provides a way to quickly create preliminary droplet generation structures in thermoplastic materials. However, both techniques have certain limitations in achieving highly integrated complex droplet systems, especially when multilayer structures or intricate three-dimensional geometries are required.

### 4.3. Application of Integration Devices

Compared with continuous flow mixing technology, microdroplet mixing technology has obvious advantages. Each droplet can be regarded as a separate reaction unit, which reduces mutual contamination when a large number of parallel or continuous reactions are carried out, and discrete droplets require less reagent consumption. Notably, the utilization of internal droplet motion and wall shear interaction can effectively solve the problems of flow-induced delamination and mixing difficulties. Therefore, the application of microdroplet mixing technology in the field of life sciences and analytical chemistry is highly promising. Micromixers within droplet generators enable precise control over polymerization and nanoparticle synthesis, allowing for tailoring of particle size, morphology, and composition [22]. This is crucial for creating materials with specific properties for drug delivery, imaging, and other applications.

Efficient and rapid mixing within droplets is crucial for accelerating reaction kinetics and reducing overall reaction time. To enhance internal mixing and thus improve reaction efficiency, Ismagilov et al. reported a substantial increase in the mixing rate of reagents when droplets flowed through curved channels. They implemented this by designing specific channel sections featuring multiple U-shaped segments. As droplets traverse these U-shaped structures, the resulting accelerated internal flow disrupts the laminar regime, leading to thorough homogenization of the different reagents or samples contained within the droplet. Effective mixing within droplets is a fundamental requirement in lab-on-chip systems for various experimental processes, such as sample pre-treatment, dilution, chemical reactions, and the supply of cell culture media [168]. Achieving adequate mixing of two droplets after merging, however, requires further consideration [169]. As shown in Figure 14c, the droplets contain different reagents, which are mixed in the bend region to accomplish the control and detection of chemical reactions. Liquid motion in conventional microfluidics is characterized by laminar flow, which makes liquid mixing difficult. In contrast, cyclic motion can provide the highest droplet merging efficiency by inducing a stretch-folding mode [170]. A study describes a novel asymmetric flow-focused droplet generator that enables enhanced mixing during the formation of droplets in the droplet-forming region of a microfluidic device under the effect of 2D- or 3D-dimensional asymmetric eddy currents [171]. This droplet generator simplifies the design of microfluidic devices by enabling both droplet formation and rapid mixing of reagents within the droplet. Table 4 shows the summary of integrated systems.

The integration of micromixers and droplet generators extends beyond simple mixing enhancement, offering a range of powerful applications as follows.

**Figure 16:**
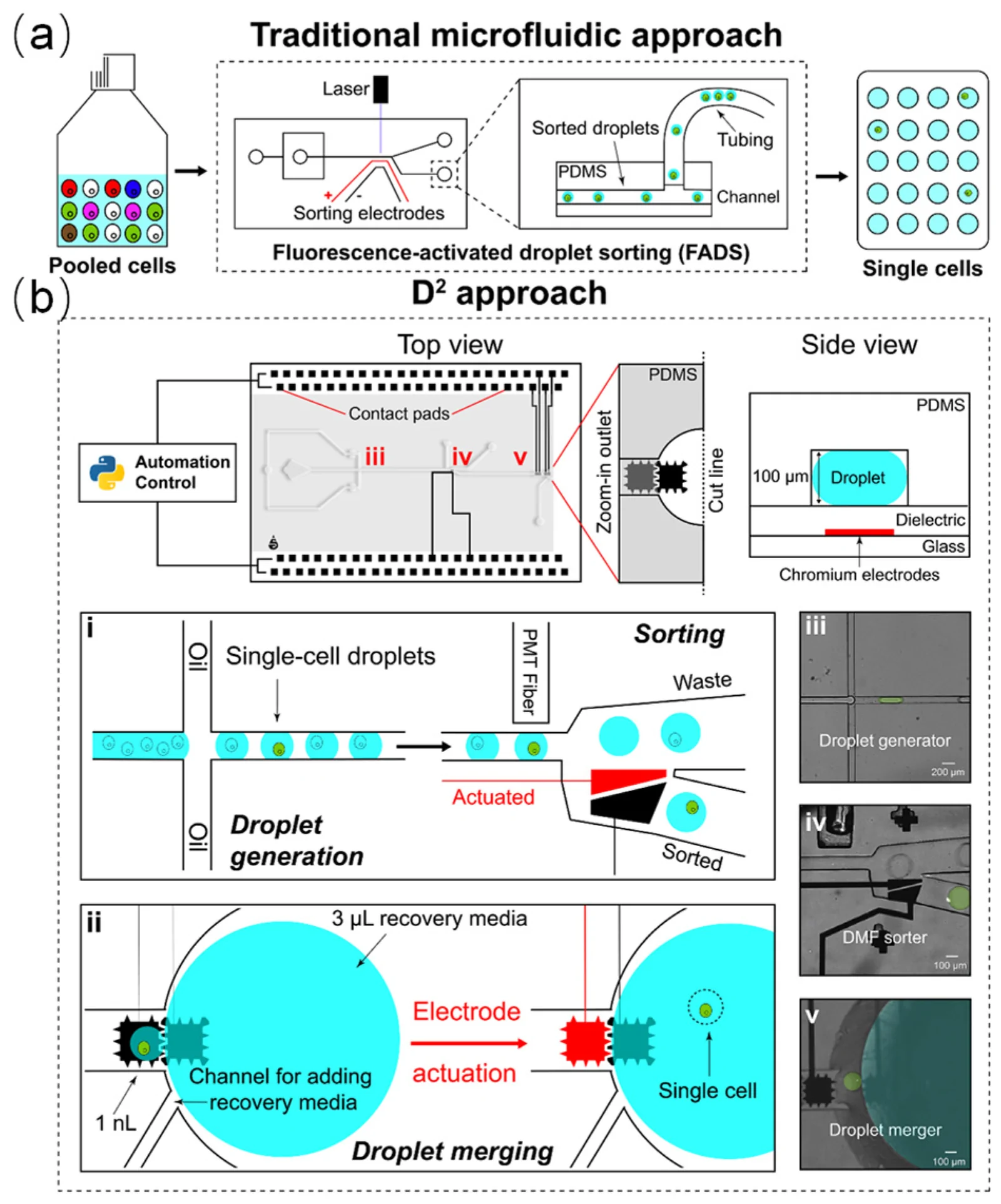
High-throughput single-cell activity-based screening and sequencing of antibodies using droplet microfluidics. (**a**) Traditional droplet microfluidic system for sorting/recovering single-cell targets from a pooled cell population. (**b**) D^2^ device. The contact pads are connected to the in-house automation control system (top view). Reprinted with permission from Deng et al., Lab Chip 25, 4410 (2025) [167]. Copyright 2025 Royal Society of Chemistry.

(1) High-throughput screening and assays: Droplet compartmentalization, combined with efficient mixing, creates ideal platforms for high-throughput screening of chemical libraries (Figure 16), drug candidates, and biological samples [7]. This minimizes cross-contamination and enables parallel analysis of thousands of individual reactions, such as drug screening, DNA analysis, immunoassays, and cell-based assays [172].
(2) Single-cell analysis and multi-omics: In recent years, single-cell analysis utilizing multi-technology microchips has profoundly impacted numerous research fields. Among its applications, high-throughput single-cell sequencing has received significant attention [115,173,174]. Droplets act as individual microreactors for isolating and analyzing single cells, allowing for high-throughput studies of cellular heterogeneity, gene expression, protein analysis, epigenetic analysis, and other cellular processes at the single-cell level [175,176]. This technology is transforming fields such as cancer biology, immunology, and developmental biology. Integrating downstream multi-omics analyses (genomics, transcriptomics, proteomics) within the droplet workflow provides a comprehensive view of cellular states and dynamics.
(3) Advanced material synthesis: Precise control over mixing and reaction conditions within droplets enables the synthesis of complex materials with tailored properties, such as Janus particles, core–shell particles, microgels, and other microstructured materials. This opens up new possibilities for designing and synthesizing advanced materials with unique functionalities. The micromixer described in one study is capable of producing liposomes as small as 24 nm with monodisperse liposome clusters at productivity rates up to 41 mg/h [177].
(4) Drug delivery and formulation: Droplet microfluidics, enhanced by micromixers, can encapsulate drugs, therapeutic proteins, or other biomolecules within microparticles or nanoparticles for controlled drug release and targeted delivery. Precise control over mixing and droplet size optimizes drug loading and release profiles [178].
(5) Microreactors for chemical and biological studies: Droplets function as individual microreactors, offering precise control over reaction parameters and minimizing reagent consumption. This is invaluable for studying reaction mechanisms, optimizing reaction conditions, and conducting small-scale chemical and biological experiments.
(6) Droplet manipulation and merging for dynamic studies: Integrating droplet generation with micromixers and droplet manipulation techniques (e.g., merging, splitting) allows for studying dynamic processes, such as cell–cell interactions, chemical reactions triggered by merging different droplets, and time-dependent analyses of biological systems [179].

## 5. Challenges and Future Perspectives

Droplet microfluidics has emerged as a powerful tool in various fields, including biology, chemistry, and medicine, enabling high-throughput experimentation, single-cell analysis, and material synthesis at the microscale. Despite remarkable advancements, several challenges and limitations hinder its broader application and widespread commercialization. This overview examines the key technical hurdles, promising research directions, and future perspectives of droplet microfluidics.

### 5.1. Material Science and Microfabrication

Current microfluidic chip fabrication heavily relies on PDMS and soft lithography. While versatile, PDMS suffers from drawbacks such as swelling in organic solvents, absorption of small hydrophobic molecules, and difficulty in mass production. In a single-function device, material choice is straightforward. In an integrated device, it becomes a complex compromise. The optimal material for a micromixer may not be ideal for a droplet generator on the same chip. The key challenge is managing dissimilar requirements within a monolithic device. Future research could focus on the following points.

#### 5.1.1. Alternative Materials

A critical challenge is the creation of heterogeneous surface properties. For example, an upstream aqueous mixer section benefits from hydrophilic surfaces to minimize protein adsorption, while the downstream droplet generation junction requires highly hydrophobic surfaces to ensure clean, oil-wetted pinch-off (i.e., for water-in-oil droplet formation) and prevent droplet shearing against the walls. Achieving this on a continuous, bonded chip requires advanced fabrication techniques.

The chosen material must be chemically inert to the entire sequence of reagents used in the integrated workflow. This includes not only the primary reactants but also the continuous oil phase, surfactants, and solvents used in mixing. Materials such as PDMS, while excellent for rapid prototyping, suffer from swelling in organic solvents and adsorption of small molecules, compromising the integrity of multi-step, integrated assays [180]. Consequently, there is a need to transition to robust materials such as glass or thermoplastics for clinical-grade integrated systems.

#### 5.1.2. Advanced Fabrication Techniques

Compared to the development of single-function chips, the fabrication of integrated devices is more challenging. The focus is on how to integrate different functional units into the same substrate. The process must ensure precise alignment between the mixer and generator, forming a robust, leak-free connection around complex geometric structures. While soft lithography excels at prototyping, transforming these precise integrated designs into mass-producible commercial products necessitates a transition to industrial-scale manufacturing methods. The production of thermoplastic materials relies on advanced processes such as injection molding and hot stamping, while high-precision 3D printing can construct truly integrated devices in a single step, enabling complex internal fluid channel designs and overcoming the limitations of traditional alignment and bonding technologies.

### 5.2. Detection and Analysis

Microfluidic chips have the advantages of high throughput and rapid generation of large numbers of droplets, but there are still challenges in real-time detection and analysis applications. Therefore, the main research areas in the future mainly include the following.

#### 5.2.1. High-Speed Imaging and Sensing

Developing high-speed, high-resolution imaging techniques and sensors compatible with droplet microfluidics, allowing for real-time monitoring of droplet contents and reactions [181].

#### 5.2.2. Integrated Detection Methods

Integrating various analytical techniques such as fluorescence, Raman spectroscopy, mass spectrometry, and electrochemical detection directly into microfluidic chips for comprehensive analysis of droplet contents [182].

#### 5.2.3. Data Acquisition and Processing

Implementing automated data acquisition and analysis pipelines for managing the vast amounts of data generated by high-throughput droplet experiments. Machine learning and artificial intelligence can play a crucial role in data interpretation and pattern recognition [91].

### 5.3. Droplet Control and Manipulation

There are still some outstanding issues that need to be addressed in order to generate droplets accurately and efficiently. In passive generation, it is crucial to design a system that can stabilize the nozzle flow for a long period of time. Due to its rather low flow rate ratio (φ ≪ 1), the nozzle flow usually becomes unstable within a few minutes, owing to the variation in the syringe pump flow [29]. In a step-emulsification droplet generator, the interplay between shear-thinning fluid properties and flow conditions influences how satellite droplet size varies with operating parameters. However, the precise mechanism through which the fluid’s shear-thinning characteristics impact satellite droplet formation warrants further investigation [106]. Active droplet generators are mainly realized by adding other components or external fields to the microfluidic system. Currently, electrical, magnetic, centrifugal, optical, thermal and mechanical methods are more commonly used, whereby flow, viscosity, interfacial tension, channel wettability and fluid density can be altered by introducing electrical, magnetic and centrifugal forces. New active control techniques can be developed, such as the use of magnetorheological effects to regulate viscosity, thermosensitive surfactants to regulate interfacial tension, and magnetic wetting effects to regulate channel wettability. Thus, active control provides more flexibility and new tools for manipulating droplet formation [29]. Precise control over droplet size, generation frequency, and content is essential for various applications. Challenges remain in the following.

(1) Stable tip-streaming: Ensuring long-term stability of tip-streaming mode for generating micrometer droplets, as it is susceptible to flow rate fluctuations. Implementing constant-pressure-driven flows and advanced 3D microfluidic designs can enhance stability.
(2) Digital microfluidics (DMF): Further developing DMF technology for precise manipulation of individual droplets, enabling complex operations such as merging, splitting, and transporting droplets for sophisticated assays.
(3) Handling complex fluids: Expanding droplet microfluidics to handle non-Newtonian fluids, such as shear-thinning and viscoelastic fluids, which are relevant to many biological and industrial applications. Understanding the influence of fluid properties on droplet formation and stability is crucial.

### 5.4. Single-Cell and Multi-Omics Analysis

Although droplet microfluidics has made significant progress thanks to the efforts of many researchers, limitations in processing, chip structures, and control of external fields have restricted its wide application in fields such as biology and medicine. During the process of generating droplets in microfluidic devices, cell survival is relatively low, and the shear stress during droplet separation can slightly damage cells [183]. It is still very challenging to precisely control the number of cells in the droplet as well as cell escape [184,185]. Droplet microfluidics has revolutionized single-cell analysis, enabling high-throughput studies of cellular heterogeneity. Future directions include the following.

(1) Improved cell viability: Minimizing shear stress and optimizing droplet generation parameters to maintain high cell viability during encapsulation and processing, particularly for delicate cell types.
(2) Precise cell encapsulation: Developing techniques for precise control over cell loading in droplets, including encapsulating specific numbers and combinations of cells for co-culture studies and heterogeneous tissue engineering.
(3) Multi-omics integration: Combining multiple single-cell omics analyses, such as genomics, transcriptomics, proteomics, and metabolomics, within the same droplet for a comprehensive understanding of cellular processes and interactions.
(4) Data integration and analysis: Developing bioinformatics tools and platforms for integrating and analyzing complex multi-omics datasets generated from single-cell droplet experiments.

### 5.5. From Academic Innovation to Clinical Reality

There exists a gap between the innovative, complex integrated designs in academic literature and their translation into medical diagnostic tools and other high-throughput applications. For example, although ddPCR is recognized as a powerful tool for nucleic acid detection [186,187]. most integrated microfluidic devices used for it have not yet been regulatively approved. This can be attributed to several challenges:

(1) Robustness with clinical samples: The intricate microchannels are prone to clogging by particulates in real-world patient samples, leading to high failure rates.
(2) Material limitations: The prevalent use of PDMS leads to the adsorption of essential PCR reagents, compromising the accuracy and reproducibility required for clinical decision-making.
(3) Incomplete workflow integration: An effective diagnostic tool must be capable of providing a “sample-to-answer” solution. Most designs primarily focus on the ddPCR step, neglecting the upstream sample preparation, downstream detection, or sophisticated liquid handling required for full automation, which is a major pragmatic bottleneck.

Furthermore, despite active academic research and development into various simplified generation techniques, the widespread adoption of straightforward generation technologies for point-of-care diagnostics faces multifaceted challenges. These hurdles primarily stem from the high development costs and stringent regulatory approvals, the inherent trade-off between simplicity and performance (potentially compromising sensitivity or specificity), difficulties in achieving scalable manufacturing and cost control, resistance from established infrastructure and user habits, as well as market fragmentation and the complexity of integration with digital health ecosystems. Collectively, these factors impede the transition of many simplified technologies from laboratory settings to broad commercial application. For instance, a significant translational hurdle is the exceptionally high throughput required for detecting rare mutations in applications such as liquid biopsy. This necessitates the generation of tens of millions of droplets from milliliter-scale samples, a volume at which many intricate, multi-component microfluidic devices frequently encounter issues with long-term stability and susceptibility to clogging.

Beyond medical diagnostics, one of the important applications where droplet generation with controlled mixing is crucial is high-throughput drug screening using droplet microfluidics. However, this field faces its own set of significant challenges. Achieving truly high-throughput screening demands the generation of tens of millions of droplets, often encapsulating single cells or beads, from milliliter-scale samples. Many complex, multi-component microfluidic devices struggle with the long-term stability and consistent droplet generation and merging required at this scale. Furthermore, precisely controlling the mixing kinetics within each pico- or nanoliter droplet, managing cell viability and culture conditions, and developing robust methods for high-content analysis post-encapsulation remain critical hurdles. The academic focus on novel device architectures often overlooks the practical aspects of sample variability, assay multiplexing, and the integration of sophisticated detection systems compatible with droplet arrays.

Therefore, the pathway to clinical approval lies not in creating even more complex designs, but in solving these fundamental challenges of robustness, materials science, and full workflow automation. Future perspectives in droplet microfluidic high-throughput drug screening will necessitate the development of more robust microfluidic platforms capable of handling diverse biological samples. This will also involve the exploration of novel materials with minimal adsorption and enhanced biocompatibility. Ultimately, the seamless integration of all workflow steps, from sample introduction to data analysis, will be crucial for enabling more reliable, reproducible, and efficient drug discovery.

### 5.6. Interdisciplinary Collaboration

Further advancements in droplet microfluidics necessitate interdisciplinary collaborations between engineers, biologists, chemists, physicists, and computer scientists. Integrating expertise in microfabrication, fluid dynamics, optics, analytical chemistry, and bioinformatics is essential for overcoming current limitations and realizing the full potential of this technology.

Particularly, the convergence of microfluidics with AI and ML presents a powerful pathway to overcome many of these challenges. For instance, in the realm of optimized design, ML models are being employed for the inverse design of complex fluidic networks, rapidly iterating to find optimal geometries for mixing or droplet stability that are non-intuitive for human designers. Furthermore, deep learning offers promising analytical tools for processing the large datasets generated by high throughput integrated systems, though its practical application still depends on careful consideration of dataset quality, prediction errors, model transferability, experimental validation, and real-time processing requirements, which are beyond the scope of this review. This emerging synergy between the “hardware” of microfluidics and the “software” of AI promises to accelerate discovery and create truly autonomous, “smart” lab-on-a-chip platforms.

In conclusion, droplet microfluidics holds immense promise for revolutionizing various scientific and technological domains. Addressing the challenges outlined above and pursuing the identified research directions will pave the way for wider adoption and commercialization of this transformative technology. The future of droplet microfluidics is bright, with potential to significantly impact healthcare, biotechnology, and materials science.

## Figures and Tables

**Table 1:** Summary of representative micromixers.

Ref.	Type	Geometry Description	Reynolds Number (Re)	Mixing Index (%)
Zhang et al. [23]	Active: AC electrothermal	Thin-film resistive heaters with asymmetric AC electrodes	–	~89
Goodarzi et al. [25]	Active: induced-charge	T-micromixer with conductive curved arc plates	–	91.86–95.44
Jahangirifard et al. [45]	Active pulsatile + deformable baffles	Deformable baffles in straight channel	1.25	92
Javaid et al. [40]	Passive	Serpentine with sinusoidal side walls	0.1–50	~95
Zoupanou et al. [51]	Passive	Spiral vs. serpentine (PMMA, 3D)	–	~87
Qin et al. [52]	Passive	T-shaped double-spiral and serpentine	1–300	97
Raza et al. [69]	Passive	Three-dimensional unbalanced SAR with circular mixing modules	20	94
Wang et al. [70]	Passive	Three-dimensional PDMS SAR (splitting–stretching–recombination)	0.01–10	>90

**Table 2:** Summary of representative droplet generators.

Ref.	Geometry Type	Droplet Size (μm)	CV (%)	Generation Frequency (Hz)
Korczyk et al. [99]	Cross-flow (T-junction)	400–1600	–	–
Cho et al. [103]	Flow-focusing (asymmetric trapezoidal cross-section)	38.2–208.2	–	–
Kalantarifard et al. [104]	Flow-focusing/co-flow	125–340	<0.2	–
Lashkaripour et al. [105]	Flow-focusing (ML-designed)	<10	<0.21	20
Wong et al. [111]	Step emulsification	140–170	Monodisperse	~32
Moon et al. [118]	Flow-focusing + pulsatile pressure	<14	Monodisperse	–
Park et al. [119]	T-junction (passive generation + SAW mixing)	10–100	–	–
Wang et al. [120]	Valve-based on-demand	916–987	–	10

**Table 3:** Comparison of integration strategies: mix-then-encapsulate vs. encapsulate-then-mix.

Aspect	Mix-Then-Encapsulate	Encapsulate-Then-Mix
Reaction initiation	Immediate after mixing, before droplet formation	Triggered at controlled time after droplet generation
Mixing uniformity	Bulk homogenization; highly uniform composition	Intra-droplet mixing; may have compositional gradients
Suitable reactions	Slow reactions; quantitative assays (e.g., ddPCR)	Fast reactions; single-cell analysis; multi-step processes
Device complexity	Lower; passive mixer upstream only	Higher; serpentine channels, electrodes, or transducers required
Droplet stability	Unaffected by upstream mixing	May be affected by bending-induced deformation
Reagent compatibility	All reagents must be compatible after mixing	Reagents can remain separated until triggered

**Table 4:** Summary of integrated systems.

Ref.	Integration Strategy	Droplet Generator Type	Mixing Performance	Application
Ma et al. [146]	Encapsulate-then-mix	–	Enhanced in serpentine	Cell encapsulation, drug screening
Chung et al. [145]	Encapsulate-then-mix	Flow-focusing	In-droplet mixing via bends	Chemical reactions
Shen et al. [147]	Encapsulate-then-mix	Flow-focusing	–	Microreactors
Nan et al. [148]	Mix-then-encapsulate	Flow-focusing	Uniform mixing	Concentration gradient screening
Belousov et al. [171]	Encapsulate-then-mix	Flow-focusing	Enhanced by asymmetric eddies	Rapid reagent mixing
Deng et al. [167]	Encapsulate-then-mix	Droplet microfluidics	–	Single-cell antibody screening

## Data Availability

The data that support the findings of this study are available from the corresponding author upon reasonable request.
